# Which patients with metastatic hormone-sensitive prostate cancer benefit from docetaxel: a systematic review and meta-analysis of individual participant data from randomised trials

**DOI:** 10.1016/S1470-2045(23)00230-9

**Published:** 2023-07-01

**Authors:** Claire L Vale, David J Fisher, Peter J Godolphin, Larysa H Rydzewska, Jean-Marie Boher, Sarah Burdett, Yu-Hui Chen, Noel W Clarke, Karim Fizazi, Gwenaelle Gravis, Nicholas D James, Glenn Liu, David Matheson, Laura Murphy, Robert E Oldroyd, Mahesh K B Parmar, Ewelina Rogozinska, Patrick Sfumato, Christopher J Sweeney, Matthew R Sydes, Bertrand Tombal, Ian R White, Jayne F Tierney

**Affiliations:** MRC Clinical Trials Unit at UCL, Institute of Clinical Trials and Methodology, London, UK; MRC Clinical Trials Unit at UCL, Institute of Clinical Trials and Methodology, London, UK; MRC Clinical Trials Unit at UCL, Institute of Clinical Trials and Methodology, London, UK; MRC Clinical Trials Unit at UCL, Institute of Clinical Trials and Methodology, London, UK; Biostatistics Unit, Institut Paoli-Calmettes, Marseille, France; MRC Clinical Trials Unit at UCL, Institute of Clinical Trials and Methodology, London, UK; Department of Biostatistics and Computational Biology ECOG-ACRIN Cancer Research Group, Dana-Farber Cancer Institute, Boston, MA, USA; Department of Surgery and Department of Urology, The Christie and Salford Royal Hospitals, Manchester, UK; Department of Cancer Medicine, Institut Gustave Roussy, Paris, France; Department of Medical Oncology, Institut Gustave Roussy, Paris, France; Institute of Cancer Research, London, UK; Department of Urology, Department of Medicine, University of Wisconsin Carbone Cancer Center, University of Wisconsin School of Medicine and Public Health, Madison, WI, USA; MRC Clinical Trials Unit at UCL, Institute of Clinical Trials and Methodology, London, UK; MRC Clinical Trials Unit at UCL, Institute of Clinical Trials and Methodology, London, UK; MRC Clinical Trials Unit at UCL, Institute of Clinical Trials and Methodology, London, UK; MRC Clinical Trials Unit at UCL, Institute of Clinical Trials and Methodology, London, UK; MRC Clinical Trials Unit at UCL, Institute of Clinical Trials and Methodology, London, UK; Biostatistics Unit, Institut Paoli-Calmettes, Marseille, France; SAiGENCI, University of Adelaide, Adelaide, SA, Australia; MRC Clinical Trials Unit at UCL, Institute of Clinical Trials and Methodology, London, UK; Institut de Recherche Clinique, Université Catholique de Louvain, Louvain-la-Neuve, Belgium; MRC Clinical Trials Unit at UCL, Institute of Clinical Trials and Methodology, London, UK; MRC Clinical Trials Unit at UCL, Institute of Clinical Trials and Methodology, London, UK

## Abstract

**Background:**

Adding docetaxel to androgen deprivation therapy (ADT) improves survival in patients with metastatic, hormone-sensitive prostate cancer, but uncertainty remains about who benefits most. We therefore aimed to obtain up-to-date estimates of the overall effects of docetaxel and to assess whether these effects varied according to prespecified characteristics of the patients or their tumours.

**Methods:**

The STOPCAP M1 collaboration conducted a systematic review and meta-analysis of individual participant data. We searched MEDLINE (from database inception to March 31, 2022), Embase (from database inception to March 31, 2022), the Cochrane Central Register of Controlled Trials (from database inception to March 31, 2022), proceedings of relevant conferences (from Jan 1, 1990, to Dec 31, 2022), and ClinicalTrials.gov (from database inception to March 28, 2023) to identify eligible randomised trials that assessed docetaxel plus ADT compared with ADT alone in patients with metastatic, hormone-sensitive prostate cancer. Detailed and updated individual participant data were requested directly from study investigators or through relevant repositories. The primary outcome was overall survival. Secondary outcomes were progression-free survival and failure-free survival. Overall pooled effects were estimated using an adjusted, intention-to-treat, two-stage, fixed-effect meta-analysis, with one-stage and random-effects sensitivity analyses. Missing covariate values were imputed. Differences in effect by participant characteristics were estimated using adjusted two-stage, fixed-effect meta-analysis of within-trial interactions on the basis of progression-free survival to maximise power. Identified effect modifiers were also assessed on the basis of overall survival. To explore multiple subgroup interactions and derive subgroup-specific absolute treatment effects we used one-stage flexible parametric modelling and regression standardisation. We assessed the risk of bias using the Cochrane Risk of Bias 2 tool. This study is registered with PROSPERO, CRD42019140591.

**Findings:**

We obtained individual participant data from 2261 patients (98% of those randomised) from three eligible trials (GETUG-AFU15, CHAARTED, and STAMPEDE trials), with a median follow-up of 72 months (IQR 55–85). Individual participant data were not obtained from two additional small trials. Based on all included trials and patients, there were clear benefits of docetaxel on overall survival (hazard ratio [HR] 0·79, 95% CI 0·70 to 0·88; p<0·0001), progression-free survival (0·70, 0·63 to 0·77; p<0·0001), and failure-free survival (0·64, 0·58 to 0·71; p<0·0001), representing 5-year absolute improvements of around 9–11%. The overall risk of bias was assessed to be low, and there was no strong evidence of differences in effect between trials for all three main outcomes. The relative effect of docetaxel on progression-free survival appeared to be greater with increasing clinical T stage (p_interaction_=0·0019), higher volume of metastases (p_interaction_=0·020), and, to a lesser extent, synchronous diagnosis of metastatic disease (p_interaction_=0·077). Taking into account the other interactions, the effect of docetaxel was independently modified by volume and clinical T stage, but not timing. There was no strong evidence that docetaxel improved absolute effects at 5 years for patients with low-volume, metachronous disease (–1%, 95% CI –15 to 12, for progression-free survival; 0%, –10 to 12, for overall survival). The largest absolute improvement at 5 years was observed for those with high-volume, clinical T stage 4 disease (27%, 95% CI 17 to 37, for progression-free survival; 35%, 24 to 47, for overall survival).

**Interpretation:**

The addition of docetaxel to hormone therapy is best suited to patients with poorer prognosis for metastatic, hormone-sensitive prostate cancer based on a high volume of disease and potentially the bulkiness of the primary tumour. There is no evidence of meaningful benefit for patients with metachronous, low-volume disease who should therefore be managed differently. These results will better characterise patients most and, importantly, least likely to gain benefit from docetaxel, potentially changing international practice, guiding clinical decision making, better informing treatment policy, and improving patient outcomes.

**Funding:**

UK Medical Research Council and Prostate Cancer UK.

## Introduction

Adding docetaxel to androgen deprivation therapy (ADT) has been shown to improve survival in patients with metastatic, hormone-sensitive prostate cancer,^1^ but uncertainty remains about precisely which patients benefit more and which benefit less. The addition of other therapies to ADT, such as the second-generation androgen receptor signalling inhibitors abiraterone, enzalutamide, and apalutamide, or radiation to the prostate (for those with low numbers of metastases), have been shown to prolong life and have become available to treat people with metastatic, hormone-sensitive prostate cancer. Therefore, it is now crucial to establish reliably who benefits most from each of these treatments.

Results of the CHAARTED^2,3^ and GETUG-AFU15^4,5^ trials and a meta-analysis of aggregate data from these trials^6^ have suggested that the volume of metastatic disease, determined by conventional imaging, modifies the effects of docetaxel, with improved survival seen in patients with a high volume of metastases, but not in those with a low volume of metastases. This finding did not appear to be borne out by the individual results of the STAMPEDE trial (confined to the cohort with metastatic disease^7^). Additionally, the timing of metastatic disease diagnosis is thought to be an additional predictor of docetaxel effects. In particular, patients with metachronous low-volume metastatic disease are posited to attain little benefit from docetaxel.^2,8^ Unsurprisingly, uncertainty about how to use docetaxel remains, with little consensus regarding the value of docetaxel even for patients with high-volume metastatic, hormone-sensitive prostate cancer.^9^ Although docetaxel might have been largely superseded by new-generation androgen receptor signalling inhibitors for intensification of ADT (doublet therapy), recent results on triple therapy—combining ADT with both an androgen receptor signalling inhibitor and docetaxel—means that the question of who benefits from docetaxel remains highly topical.^10^

The best way to assess any remaining uncertainties about the effects of docetaxel thoroughly and reliably is through the collection, checking, and rigorous re-analysis of individual participant data from relevant trials. A meta-analysis based on individual participant data can improve the quantity and quality of data available,^11,12^ thereby providing greater power than any one trial and circumventing the biases and other limitations associated with the traditional aggregate data approach.^13^ Individual participant data also allow more flexible and detailed analyses, including the ability to more thoroughly and appropriately investigate potential treatment effect modifiers.^11,12^ Therefore, we conducted this STOPCAP collaborative individual participant data meta-analysis to provide up-to-date estimates of the overall effects of adding docetaxel to ADT for patients with metastatic, hormone-sensitive prostate cancer, ascertain more precisely whether these effects vary according to prespecified characteristics of the patients or their tumours, and guide treatment choices for patients, clinicians, and policy makers.

## Methods

### Search strategy and selection criteria

In this systematic review and individual participant data meta-analysis, studies were eligible for inclusion if they were randomised controlled trials that compared ADT plus docetaxel (intervention) with ADT alone (comparator). They should have aimed to randomly assign people who were either diagnosed with de-novo (ie, synchronous), metastatic, hormone-sensitive prostate cancer or who developed metastatic, hormone-sensitive prostate cancer after previously being diagnosed with localised disease (ie, metachronous), and who were either starting or responding to first-line hormone therapy. Docetaxel could have been co-administered with supportive treatments. We did not limit inclusion criteria by outcomes assessed in the trials.

Eligible trials were identified through systematic searches conducted routinely for the STOPCAP programme of systematic reviews and meta-analyses in metastatic, hormone-sensitive prostate cancer, using an approach that has been reported previously.^1,14,15^ The literature review was done in accordance with the Preferred Reporting Items for Systematic Reviews and Meta-analyses (PRISMA) guidelines. In brief, we ran comprehensive search strategies (appendix pp 2–5) for MEDLINE (from database inception to March 31, 2022), Embase (from database inception to March 31, 2022), and the Cochrane Central Register of Controlled Trials (from database inception to March 31, 2022). We also regularly searched the clinical trials register ClinicalTrials.gov from database inception to March 28, 2023, and screened the proceedings and abstracts of relevant conferences (from Jan 1, 1990, to Dec 31, 2022) using a range of relevant search terms (including “metastatic hormone-sensitive prostate cancer”, “chemotherapy”, and “docetaxel”) and by manually searching reference lists of relevant trial reports and review articles. Eligibility was determined by three authors (LHR, SB, and CLV) with any conflicts resolved by consensus, involving other members of the core research team or advisory group as needed.

We sought individual participant data from study investigators or relevant repositories for all randomly assigned patients from eligible trials. These data were collected according to a detailed data dictionary (available on request to the corresponding author, CLV). In summary, we requested information on baseline characteristics including age, height, weight, WHO performance status, prostate-specific antigen (PSA) measurements, Gleason score, and Tumour, Node, Metastasis (TNM) stage at the time of metastatic diagnosis, along with date of randomisation and treatment allocations, date of diagnosis (both for previous localised disease, if applicable, and of metastatic disease), and number and location of metastases. We also sought information on treatments, including the type and duration of ADT, dosing schedules, and dates of commencement, and on progression and survival outcomes. We requested data based on the most recent follow-up to maximise information, and therefore power, and to allow reporting of outcomes in the longer term. Individual participant data from each trial were assessed for completeness and harmonised as far as possible, and the variables were checked for consistency, validity, and range by two authors (LHR and PJG), in discussion with core research team members (CLV, DJF, and JFT). Any issues arising were queried and resolved through contact with the trial teams.

Procedures for secure transfer and storage of deidentified individual participant data were set out in an approved ethics application (UCL Research Ethics Committee Project identifier 14095/001). Data use agreements ensured that investigators complied with all local laws and statutes applicable to the performance of clinical studies, and that the individual participant data were transferred according to relevant Data Protection Laws.

Unless otherwise stated, all methods were prespecified in a protocol, first registered in PROSPERO in July, 2019, and updated in August, 2021, in concert with the development of a detailed statistical analysis plan.

For the **protocol and statistical analysis** plan see http://www.stopcapm1.org/protocol/

### Outcomes

For analyses of the overall effects of docetaxel, the primary outcome measure was overall survival, defined as the time from randomisation until death from any cause. Patients known to be alive (including those lost to follow-up) were censored on the date of the most recent follow-up. Secondary outcome measures were progression-free survival (defined as the time from randomisation until first clinical or radiological progression or death from any cause, whichever occurred first) and failure-free survival (defined as time from randomisation until first biochemical, clinical, or radiological progression or death from any cause, whichever occurred first). For the secondary outcome measures, patients alive and without progression were censored on the date of the last follow-up. We also planned to assess the effects of adding docetaxel to ADT on additional, sensitivity outcomes of radiological progression-free survival, prostate cancer-specific survival, time to PSA failure, and time to castrate resistance.

For each trial, the risk of bias (low risk, some concerns, or high risk) was assessed using version 2 of the Cochrane Risk of Bias Assessment Tool.^16^ These assessments were based on the main outcomes of overall survival, progression-free survival, and failure-free survival, and for the randomisation process, deviations from the intended intervention, missing outcome data, and measurement of the outcome. The selection of the reported trial result domain is not relevant for an individual participant data meta-analysis but was included for completeness. The assessments were based on information from trial protocols and manuscripts, information from trialists, and direct checks of the individual participant data. The direct checks were used to explore patterns of treatment allocation, the balance of baseline characteristics by treatment group, the degree of missing outcome data, how outcomes were measured, and the balance of follow-up. Risk-of-bias assessments were done independently by two authors (SB and LHR), with disagreements resolved through discussion with a third author (JFT).

### Data analysis

Unless otherwise stated, all analyses were prespecified in the protocol and statistical analysis plan, and all randomly assigned participants were included on an intention-to-treat basis. All p values are two-sided. Initially, we carried out investigations at the trial level in preparation for pairwise individual participant data meta-analysis.

To maximise power and precision, and to lessen the chance of variation in effects between trials, the analyses of each outcome were adjusted for a core set of baseline covariates within each trial,^17^ chosen a priori for their known prognostic impact or because they were likely to be available for all trials. These covariates were age, PSA concentration, WHO performance status, Gleason sum score (all at randomisation), and whether the patient had been diagnosed with synchronous or metachronous metastatic disease. Mean imputation (which has been shown to be preferable to multiple imputation for baseline covariates in randomised trials^18,19^) was applied separately for each trial to account for any missing data in the adjustment factors, to ensure compliance with intention to treat, and to avoid bias. Imputation was performed once, before model fitting, and imputed data were subsequently used in all statistical analyses.

One of the eligible trials, the STAMPEDE trial, used an adaptive design^20–22^ in which the research or control treatments could change over time. Therefore, all analyses of STAMPEDE trial data were stratified for patient-level indicator variables corresponding to specific randomisation epochs—ie, to periods of time during which the design remained constant.

The proportional hazards assumption was tested using Schoenfeld residuals from Cox regression, plotted against log time.^23^ We also visually assessed the proportionality of hazards with Kaplan-Meier survival curves and by plotting predicted time-varying hazard ratio (HR) functions from flexible parametric models with time-varying effects applied to treatment effect and trial membership. Assessments were made for each outcome, for each trial separately and across trials. We made extensive use of analysis methods designed to take account of non-proportionality of hazards.

We prespecified the following covariates for consideration as potential effect modifiers: age, WHO performance status, clinical T stage, nodal involvement, Gleason sum score, baseline alkaline phosphatase concentration, timing of metastatic disease (synchronous or metachronous), lymph node disease only, location of metastases (bone only, visceral only, and bone and visceral), number of bone metastases, BMI, risk group (LATITUDE trial definition,^24^ with high risk defined by the presence of any two of the following: Gleason sum score ≥8; at least three bone metastases; and any visceral metastases), and volume of disease (at least four bone metastases, any visceral metastasis, or both, dropping the criterion for one lesion beyond pelvic bones and vertebral column used in the CHAARTED trial^2^). However, we restricted analyses of interactions between treatment and patient-level characteristics to those covariates with sufficient data and with sufficient power to detect an effect.^25^ Because our primary analysis model was fixed-effect, we approximated power by considering all data as though coming from a single trial^26^ and applying the formula of Schmoor and colleagues.^27^ We prespecified progression-free survival, which includes both clinical and radiological progressions and deaths, as the main outcome for these analyses to maximise the information size and power.

Our primary analysis approach was to pool adjusted HR estimates of the effect of docetaxel for each trial in a two-stage, fixed-effect, inverse-variance meta-analysis. χ^2^ heterogeneity tests and the *I*^2^ statistic^28^ were used to assess statistical heterogeneity of effects across trials.

If evidence of non-proportional hazards was detected, we used the HR and log-rank test as the primary estimate of effect and statistical test but placed greater inferential emphasis on absolute outcome differences at 5 years. To estimate absolute survival, we fitted a flexible parametric survival model^29^ to all data, adjusting for trial membership and the core (imputed) covariate set. When analysing treatment–covariate interactions, models were formulated to avoid aggregation bias.^30^ Time-varying effects were placed on trial membership and treatment parameters (including treatment interactions where appropriate) to allow trial-specific baseline hazard functions and to account for non-proportionality of hazards between treatment groups. Smoothed patient-averaged (marginal) survival curves were estimated using regression standardisation^29,31^ across observed covariate values, using the standsurv package^32^ in Stata, enabling absolute differences with 95% CIs to be derived.

We conducted sensitivity analyses to test the robustness of the results from the primary analysis model, based on the DerSimonian and Laird^33^ random-effects model, a complete-case adjusted model, and unadjusted estimates of effect.

We investigated how the effects of docetaxel on progression-free survival varied by participant characteristics (covariates) only when there were sufficient data, and sufficient power for analyses. If data were insufficient or markedly unevenly distributed across subgroup categories, we collapsed or re-categorised them for analysis. Wherever tests for interaction on the progression-free survival outcome were found to be significant at the 10% level, we proceeded to conduct similar analyses based on overall survival. As a sensitivity analysis, we performed a post-hoc assessment of type I error due to multiplicity, using the Hochberg procedure^34^ with α set at 0·10.

We used Cox regression to estimate treatment-by-covariate interactions within each trial, adjusting for the key covariates described above. Within-trial interaction HRs were combined using a fixed-effect, two-stage meta-analysis.^30,35^ Subgroup-specific HRs were also estimated on the basis of within-trial information to avoid aggregation bias.^36^

If multiple covariates were found to interact significantly (p<0·10) with the effects of docetaxel on progression-free survival, we assessed the strength of each such interaction effect independently of the others in an exploratory analysis, by fitting a one-stage meta-analysis model to all trial data simultaneously. We used an adjusted Cox regression containing main effects of treatment and of each covariate found to modify treatment, plus treatment-interaction terms for these covariates, structured appropriately to avoid aggregation bias.^26^ Additionally, if two or more interactions of particular significance or clinical interest were identified, we derived a new covariate formed from the cross-tabulation of the two subgroup variables and estimated treatment effects within each such category. To account for missing data imputation, imputed categorical covariates were treated as continuous within the model, but subsequent testing and effect estimation was performed conditionally on the set of observed (non-imputed) covariate values. We also did exploratory statistical hypothesis testing based on Cox regression models to test whether any identified treatment–covariate interactions were explained by the effects of any other covariate or combination of covariates.

To estimate the absolute survival difference at 5 years within covariate subgroups, we again fitted a flexible parametric model with an appropriate one-stage modelling structure to prevent aggregation bias.^26^ Absolute treatment differences were then obtained by regression standardisation over observed covariate values. All analyses were done with Stata (version 17.1)

This study is registered with PROSPERO, CRD42019140591.

### Role of the funding source

The funders had no role in study design, data collection, data analysis, data interpretation, decision to submit for publication, or preparation of the manuscript.

## Results

We identified five eligible trials; we purposely did not seek individual participant data from two unpublished trials (Pedley et al^37^ and NCT00796458), previously identified through our systematic review of aggregate data,^1^ because we estimated they would comprise only around 2% of known randomly assigned participants and would therefore contribute very little to the evidence base. However, we sought and obtained data on all 2261 participants included in the three largest trials of docetaxel—GETUS-15,^4,5^ CHAARTED,^2^ and STAMPEDE^7,20^—comprising an estimated 98% of all participants randomly assigned in relevant trials (appendix p 52).

The trials recruited patients between October, 2004, and March, 2013, and accrued 385 patients (GETUG-AFU15), 790 patients (CHAARTED), and 1086 metastatic patients (STAMPEDE) to eligible comparison groups (table 1). All three trials achieved their target recruitment; however, CHAARTED was reported early on the basis of the advice of the independent data monitoring committee after the prespecified criteria for benefit had been met.^3^ Each trial aimed to randomly assign patients with either newly diagnosed (synchronous) metastatic disease or a previous diagnosis of localised disease who were now commencing ADT for metastatic disease (metachronous), with good performance status, and who were fit enough to tolerate docetaxel. Patients were randomly assigned to receive either ADT alone or ADT plus 75 mg/m^2^ docetaxel for either six cycles (STAMPEDE and CHAARTED) or up to nine cycles (GETUG-AFU15). Accepted forms of ADT across the trials included luteinising hormone-releasing hormone agonists or antagonists, combined androgen blockade, or surgical castration (table 1). The median follow-up across trials and across all participants was 72 months (IQR 55–85). Data supplied correspond to the most up-to-date reported analysis and follow-up for each trial.

For each trial, risk of bias was judged to be low, both for individual domains and overall, for each of the main outcomes (appendix pp 7–46, 53).

Data on age, WHO performance status, alkaline phosphatase concentration, PSA concentration, Gleason sum score, clinical T stage, nodal involvement, disease status at the time of randomisation, location of metastases, disease volume, and risk were supplied for all three trials. A large proportion of data were missing on alkaline phosphatase, clinical T stage, and nodal metastases in each of the three trials. Furthermore, there was a higher proportion of missing stage data in patients with metachronous (30% missing stage data) than for those with synchronous (19% missing stage data). From the available data, the median age at randomisation was 65 years (IQR 59–70), 1697 (75%) of 2261 participants had a WHO performance status of 0, and 1432 (63%) had a Gleason sum score of 8 or higher (table 2). Of the 1789 (79%) participants with clinical T stage recorded, most were T stage 3 or 4 at diagnosis (1173 [66%]). Across the three trials, 1883 (83%) patients were diagnosed with synchronous metastatic disease, although the proportion in STAMPEDE was higher (1036 [95%] of 1086) than for the other two trials (847 [72%] of 1175). Evidence of bone metastases was specifically reported in 1742 (77%) patients; however, a further 277 patients in the CHAARTED trial, who were reported as having low-volume disease, would by definition have either lymph node disease or three or fewer bone metastases. Based on those patients with clinical T stage recorded, in STAMPEDE, the majority were clinical T stage 3 (601 [61%] of 985) or 4 (245 [25%]), whereas 11 (6%) of 183 patients in GETUG-AFU15 and 79 (13%) of 608 patients in CHAARTED were T stage 4. Finally, based on those patients for whom it was recorded, the proportion of participants with involved nodes at randomisation was similar across all trials (table 2).

The final set of adjustment factors were age (as reported; no transformations or imputation); PSA (log transformed; five missing values imputed); WHO performance status (1 *vs* 0; 19 missing values imputed); Gleason sum score (6 *vs* else, 7 *vs* else, 8 *vs* else, 9 *vs* else, 10 *vs* else; 222 missing values imputed), and timing of metastatic disease status (synchronous *vs* metachronous; six missing values imputed). Additionally, for the adaptive STAMPEDE trial we adjusted for randomisation epochs: Oct 5, 2005, to April 5, 2011 (including random assignment to zoledronic acid or celecoxib with or without zoledronic acid);^38^ April 6 to Nov 14, 2011 (including random assignment to zoledronic acid with or without docetaxel), and Nov 15, 2011, to March 31, 2013 (including random assignment to abiraterone).^39^

There was limited evidence of proportional hazards for overall survival (appendix p 54). However, there was considerable evidence of non-proportional hazards in each trial both for progression-free survival and failure-free survival (appendix p 54). Hence, for all of these outcomes, interpretation focuses on absolute differences in effect at 5 years.

Data on overall survival were available for all 2261 participants, and 1355 deaths were reported across the three trials. The adjusted analysis showed clear evidence of a relative benefit of adding docetaxel to ADT (HR 0·79, 95% CI 0·70–0·88; p<0·0001), with little evidence of statistical heterogeneity (p=0·32, *I*^2^=13%; appendix p 55). Absolute survival benefit of docetaxel was 11% (95% CI 7–15), increasing 5-year survival from 39% (36–41) with ADT alone to 49% (46–52) with ADT plus docetaxel (appendix p 56). Planned sensitivity analyses gave similar results (appendix p 47).

Data on progression-free survival were available for all 2261 participants, and 1624 events were reported across the three trials. Radiological progression (with or without a clinical progression) was the most common first event reported (1105 [68%] of 1624), followed by similar numbers of clinical progressions (264 [16%]) and deaths without evidence of progression (255 [16%]; appendix p 57). The adjusted analyses showed clear evidence of a benefit of adding docetaxel to ADT (HR 0·70, 95% CI 0·63–0·77; p<0·0001), with little evidence of statistical heterogeneity (p=0·31, *I*^2^=15%; appendix p 55). Absolute benefit of docetaxel was 9% (95% CI 6–13) on 5-year progression-free survival, increasing it from 24% (22–27) with ADT alone to 34% (31–37) with ADT plus docetaxel (appendix p 56). Results of sensitivity analyses were similar (appendix p 47).

Data on failure-free survival were available for all 2261 participants, and 1848 events were reported across the three trials. Data on failure-free survival were available for all 2261 participants and 1848 events were reported across the three trials. First events were dominated by biochemical progressions either alone (1296 [70%]) or in combination with another event (52 [3%] of 1848) and deaths without progression (87 [5%]; appendix p 58). There was evidence of a benefit of adding docetaxel to ADT (HR 0·64, 95% CI 0·58–0·71; p<0·0001), with little evidence of statistical heterogeneity (p=0·29, *I*^2^=20%; appendix p 55). Absolute benefit of docetaxel was 9% (95% CI 6–12) on 5-year failure-free survival, increasing it from 14% (13–16) with ADT alone to 23% (21–26) with ADT plus docetaxel (appendix p 56). Results of sensitivity analyses were similar (appendix p 47).

Results of analyses of the sensitivity outcomes of radiological progression-free survival, prostate cancer-specific survival, time to PSA failure, and time to castrate-resistant disease all showed clear relative benefits of adding docetaxel to ADT. The associated absolute differences in benefits were all in the region of 10% at 5 years (appendix p 48). All overall effects remained significant at 5% after post-hoc correction for multiple testing (data not shown).

**Figure 1:**
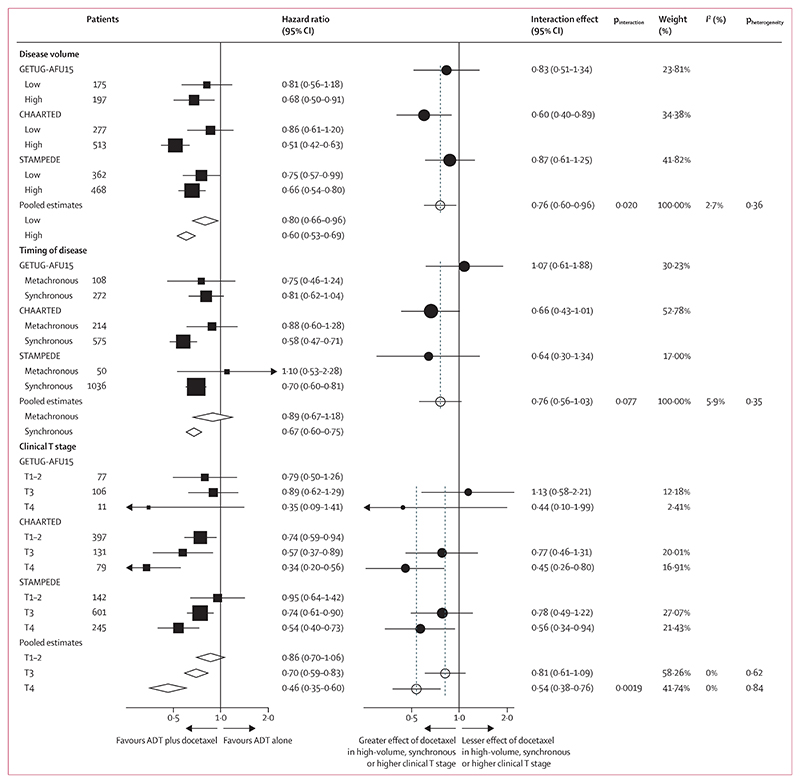
Effect of docetaxel on progression-free survival by disease volume, timing of metastatic disease diagnosis, and clinical T stage at randomisation The left-hand panel shows estimates of treatment effects within subgroups for individual trials, with boxes representing hazard ratios derived from Cox regression models fitted to each trial in turn, adjusted for the core covariate set and with missing covariate values imputed. The size of each square is directly proportional to the amount of information contributed by a trial, and the horizontal lines show the 95% CIs. The diamonds represent pooled estimates for each subgroup, derived using a within-trials framework,^36^ with the centre denoting the HR and the extremities the 95% CI. The filled circles on the right-hand panel show the interaction effects (ratio of hazard ratios) within each trial. These are derived from Cox regression models fitted to each trial in turn, including a treatment interaction term, and adjusted for the core covariate set and with missing covariate values imputed. Horizontal lines show the 95% CIs. Open circles show the meta-analysis interaction effect from two-stage, fixed-effect inverse-variance meta-analysis, with the centre denoting the hazard ratio and the extremities the 95% CI. ADT=androgen deprivation therapy.

Guided by the distribution of covariate information, and the estimated power, we investigated whether the effect of docetaxel on progression-free survival was modified by age, BMI (continuous), WHO performance status (0 *vs* 1–2), Gleason sum score (<8 *vs* ≥8), risk group (high *vs* low), disease volume (high *vs* low), clinical T stage (T0–2 *vs* T3 *vs* T4), and clinical N stage (N0 *vs* N+). Additionally, due to its clinical importance, we also included disease timing (metachronous *vs* synchronous), although power was relatively low due to subgroup imbalance. We found no evidence that the effect of docetaxel on progression-free survival was modified by age, BMI, WHO performance status, Gleason sum score, risk, or clinical N stage (appendix p 49). There was evidence that the effect of docetaxel on progression-free survival was modified by disease volume (p_interaction_=0·020), timing of metastatic disease (p_interaction_=0·077), and clinical T stage (p_interaction_=0·0019; figure 1). Clinical T stage remained significant after correction for multiple testing (Hochberg procedure with alpha 0·1 and nine subgroup interaction tests; post hoc).^34^ There was evidence that disease volume (p_interaction_=0·073) and clinical T stage (p_interaction_=0·0022) independently modified the effect of docetaxel on progression-free survival after mutual adjustment, whereas timing of metastatic disease diagnosis did not (p_interaction_=0·45; appendix p 50).

**Figure 2:**
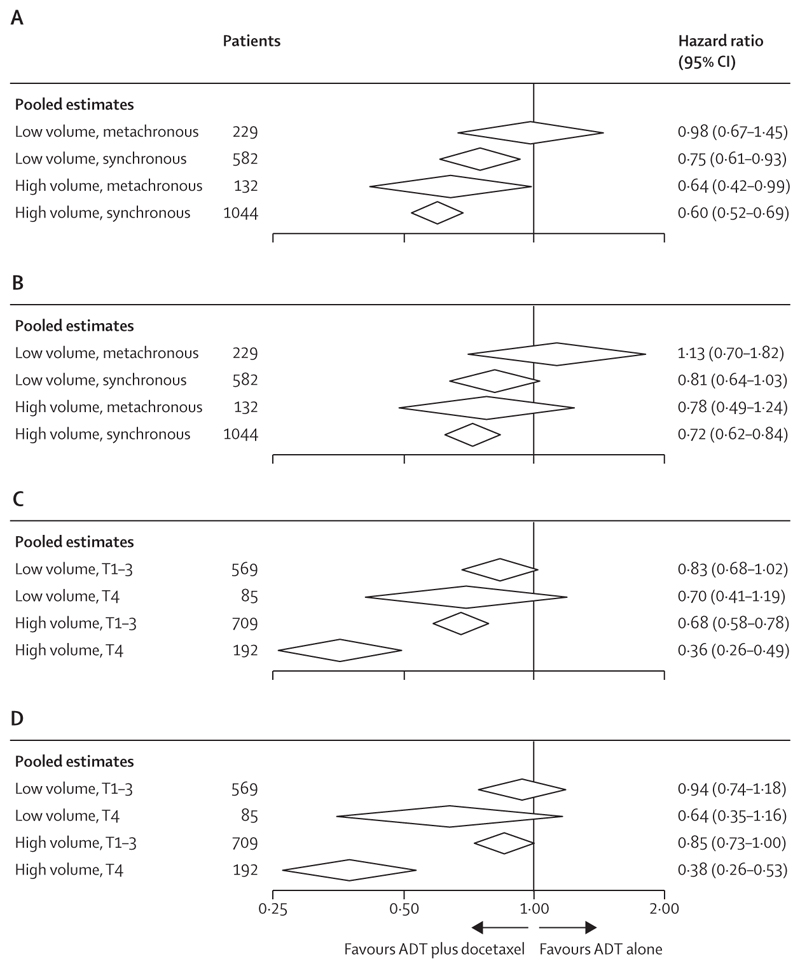
Effect of docetaxel on progression-free survival and overall survival by volume and timing of metastatic disease diagnosis and by volume and clinical T stage Relative effects of ADT plus docetaxel versus ADT alone by disease volume and timing of metastatic disease diagnosis combined on progression-free survival (A); disease volume and timing of metastatic disease diagnosis combined on overall survival (B); disease volume and clinical T stage combined on progression-free survival (C); and disease volume and clinical T stage combined on overall survival (D). Each diamond represents the pooled estimates for each subgroup, derived using a within-trials framework,^36^ with the centre denoting the hazard ratio and the extremities the 95% CI. ADT=androgen deprivation therapy.

**Figure 3:**
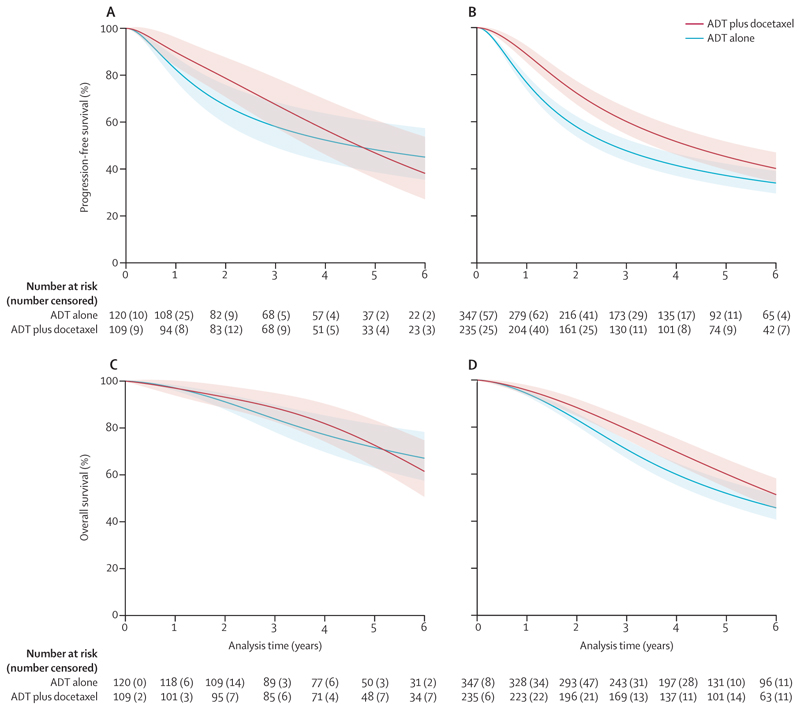
Effect of docetaxel on progression-free survival and overall survival for patients with low-volume disease, by timing of metastatic disease diagnosis Predicted survival curves for patients with low-volume metastatic disease for the subgroups of patients with synchronous and metachronous diagnosis, based on a one-stage flexible parametric meta-analysis model fitted to the entire participant sample with interaction terms between docetaxel effect and each of the four volume-by-timing subgroups, accounting appropriately for aggregation bias, adjusted for the core covariate set and with missing covariate values imputed, and using regression standardisation to estimate marginal progression-free survival curves for low-volume, metachronous disease (A); progression-free survival curves for low-volume, synchronous disease (B); overall survival curves for low-volume, metachronous disease (C); and overall survival curves for low-volume, synchronous disease (D). Red indicates ADT plus docetaxel and blue indicates ADT alone. Shaded areas denote the 95% CIs. ADT=androgen deprivation therapy.

Considering volume and the timing of the diagnosis of metastatic disease together, docetaxel did not appear to improve progression-free survival in the low-volume, metachronous disease subgroup either in relative (HR 0·98, 95% CI 0·67 to 1·45; figure 2) or absolute terms at 5 years (–1%, 95% CI –15 to 12; table 3; figure 3). Based on available data, this group almost entirely comprised patients with clinical T stages 1–3 (161 [98%] of 165). By contrast, docetaxel use was associated with a relative improvement in progression-free survival and overall survival in all other volume-by-timing subgroups (figures 2, 3; appendix p 59), with estimated absolute effects at 5 years ranging from 8% to 12% (table 3). As most participants had synchronous metastatic disease, the power to detect an interaction was relatively low.

**Figure 4:**
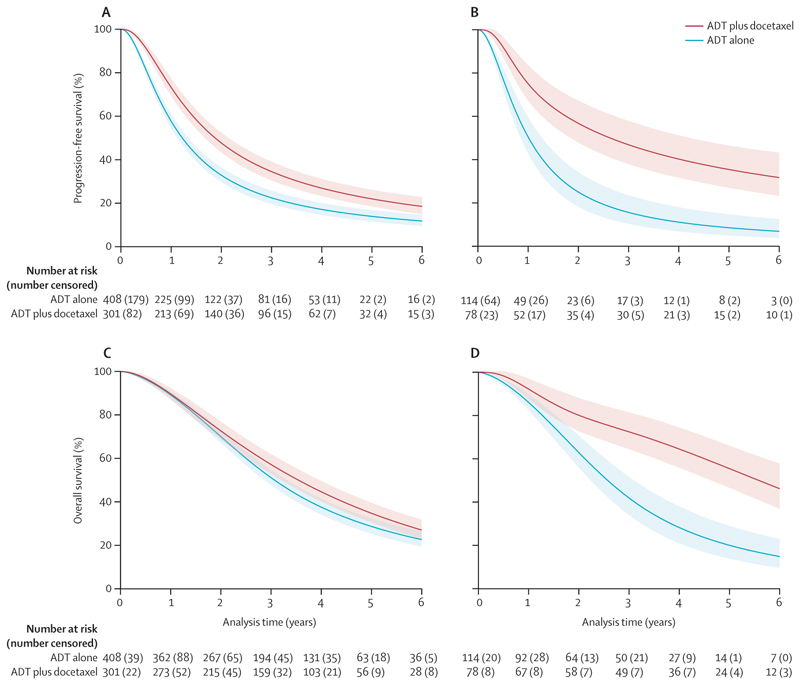
Effect of docetaxel on progression-free survival and overall survival for patients with high-volume disease, by clinical T stage Predicted survival curves for patients with high-volume metastatic disease, for subgroups based on clinical T stage, based on a one-stage flexible parametric meta-analysis model fitted to the entire participant sample with interaction terms between docetaxel effect and each of the four volume-by-stage subgroups, accounting appropriately for aggregation bias, adjusted for the core covariate set and with missing covariate values imputed, and using regression standardisation to estimate marginal progression-free survival curves for high-volume, T stage 1–3 (A); progression-free survival curves for high-volume, T stage 4 (B); overall survival curves for high-volume, T stage 1–3 (C); and overall survival curves for high-volume, T stage 4 (D). Shaded areas denote the 95% CIs. ADT=androgen deprivation therapy.

We observed a significant interaction between effect of docetaxel and T stage 4 versus other T stage categories; 5-year baseline survival rate was broadly consistent for T stage 1–2 (27%, 95% CI 22–32) and 3 (25%, 22–29) compared with T stage 4 (17%, 12–23); and the T stage 4 category correlated strongly with disease timing (326 [97%] of 335 patients with clinical T stage 4 disease also had synchronous diagnoses, regardless of volume). Therefore, patients with clinical T stages 1, 2, and 3 were collapsed into a single subgroup. Once again, we note that there is substantial missing data for clinical T stage (472 [21%]). The clearest evidence and the largest benefit of docetaxel was seen in patients with high-volume disease, who also had clinical T stage 4 disease (HR 0·36, 95% CI 0·26–0·49; figure 2): the group with the poorest prognosis (figure 4; appendix p 60). For this subgroup, the estimated absolute 5-year benefit of docetaxel on progression-free survival was 27% (95% CI 17–37), increasing it from 9% (5–15) with ADT alone to 35% (27–45) with ADT plus docetaxel (table 3). For overall survival, there was an estimated improvement in absolute survival of 35% (95% CI 24 to 47) at 5 years, improving it from 20% (14–29) with ADT alone to 55% (47–66) with ADT plus docetaxel (table 3).

Exploratory sensitivity analyses, excluding patients with metachronous disease from the analysis of volume of disease and clinical T stage combined, and similarly excluding those with clinical T stage 4 from the analysis of volume of disease and timing of diagnosis combined, and after post-hoc correction for multiple testing, gave results that were consistent with the corresponding prespecified analysis (appendix p 51).

## Discussion

Adding docetaxel to ADT-based standard care improved outcomes for patients with metastatic, hormone-sensitive prostate cancer overall, but the effects on overall survival and progression-free survival are modified by volume of metastases, timing of metastatic disease diagnosis, and clinical T stage. There was no clear evidence that docetaxel improved 5-year overall survival or progression-free survival for patients with low-volume, metachronous disease. There was, however, clear evidence of substantial improvements in 5-year overall survival and progression free survival for patients with high-volume disease, which were broadly consistent for those with metachronous and synchronous diagnoses, but greater in those with higher clinical T stage.

These results are based on harmonised individual participant data with long-term follow-up from the three largest trials, representing 98% of all patients who were randomly assigned.^2,6,7^ We meticulously checked the collated individual participant data, actively engaging with the trial teams to confirm accuracy, before applying a thorough and rigorous analytical approach. We adjusted for core covariates, accounted for missing data, assessed proportionality of hazards, and examined the consistency of overall and subgroup effects across all trials and outcomes. We have made maximum use of individual participant data to generate relative and absolute treatment effects, and to appropriately investigate interactions between treatment effects and patient characteristics, while accounting for other effect modifiers and prognostic factors. Thus, we have provided updated and more precise estimates of the overall benefits of docetaxel than have previously been reported,^1^ and corroborated associations between the effects of docetaxel, volume of metastases, and timing of metastatic disease diagnosis. Additionally, for the first time we have identified clinical T stage as a potentially important effect modifier, independent of disease volume and timing. Although we cannot be certain that this effect is unconfounded, T stage might help to further improve the characterisation of the patients most likely to gain benefit from docetaxel and, importantly, those least likely to gain benefit. These results will potentially change international practice, further guide clinical decision making, better inform treatment policy, and improve patient outcomes.

Despite the gains in power from combining trial data, our results showed no clear benefit of docetaxel for low-volume metachronous disease. As this subgroup represents less than 10% of the population in this meta-analysis, power of this subgroup analysis was limited. Volume of metastatic disease appears to strongly predict docetaxel effects; however, volume was assessed prospectively only in the CHAARTED trial and was attributed retrospectively in both GETUG-AFU15 and STAMPEDE. Although almost a quarter of patients in STAMPEDE had missing volume data, this does not appear to have affected the overall findings. We also observed that clinical T stage, assessed prospectively at baseline across the trials, strongly predicted docetaxel effects and that the effect of docetaxel on overall survival and progression-free survival was greatest for those with both high volume and clinical T stage 4. However, we recognise that with less than 20% of the included population had clinical T stage 4, and so the power of this subgroup analysis was limited. Furthermore, although this observation did not appear to be influenced by the timing of disease diagnosis, we recognise the recording of stage in those patients presenting with metachronous disease (and who are therefore likely to have initially had either surgery or radiotherapy to the primary site) might have been more inconsistent than for those presenting with synchronous metastatic disease. However, although we observed a higher proportion of missing stage data in patients with metachronous (30% missing stage data) than for those with synchronous (19% missing stage data), the distribution of other baseline characteristics was broadly consistent in patients with and without clinical T stage recorded. Therefore, we do not think that our findings are skewed by the unavailable data, and a sensitivity analysis based only on synchronous population supported the observation of greatest effect of docetaxel in those with high metastatic volume and T stage 4.

Because the participants in these trials were generally younger and had a better performance status than would typically be seen in routine clinical practice, results might not be wholly generalisable, and any decisions about use of docetaxel would also need to take into account factors such as, age, general health, and comorbid conditions.

One final limitation is that, although recently developed core outcome sets for metastatic, hormone-sensitive prostate cancer have identified important patient reported outcomes,^40^ data on outcomes such as bowel and urinary dysfunction, pain, fatigue, or sexual dysfunction were not consistently recorded or requested in this meta-analysis. Results from the individual trials have indicated that global quality of life is reduced during docetaxel treatment—most likely reflecting toxicity associated with treatment—although this does not appear to persist in the longer term.^4,41,42^

Despite evidence about the benefits of docetaxel from randomised controlled trials and a previous meta-analysis^1^ leading to changes in guidance around standard care, data suggest that docetaxel might be under-used in practice. For example, in England and Wales, only 150–200 patients per month (30–40% of those diagnosed with metastatic, hormone-sensitive prostate cancer) started docetaxel treatment in 2019–20, with numbers reduced considerably following recommendations allowing enzalutamide or abiraterone during the COVID-19 pandemic.^43^

Although all the second-generation androgen receptor signalling inhibitors (with the exception of orteronel^44^) have shown improved survival when added to standard care,^24,39,44–50^ it is less clear whether effects of these agents vary across different groups defined by baseline characteristics, or with previous or concurrent use of docetaxel. As most trials are under-powered to identify subgroup effects reliably, it is crucial to combine and analyse individual participant data from all relevant trials^24,39,44–50^ and these trials of docetaxel, to elucidate which treatment combination is preferable and for whom. Importantly these results, and the understanding of the effects of docetaxel gained through them, provide the foundation for the next stages of our ongoing STOPCAP programme, in which we aim to establish whether a doublet of ADT plus either docetaxel or an androgen receptor signalling inhibitor, or a triplet of ADT, docetaxel, and an androgen receptor signalling inhibitor is preferable and in whom.

These results delineate more clearly which patients with metastatic, hormone-sensitive prostate cancer are most likely to benefit from receiving docetaxel in addition to ADT-based therapy. This is of particular relevance now that international practice has started to move away from routine docetaxel use in this population. Notably, patients with poorer prognosis, because of high-volume disease, should be considered for docetaxel plus ADT, especially if access to other life-prolonging therapies is limited, or for triplet therapy, provided they are willing and fit enough receive it, and particularly if they also have a large primary tumour. However, outside clinical trials, clinical T stage might not be assessed routinely or is often recorded as Tx for those presenting with metastatic disease. Thus, if it is to be used to refine treatment decisions or further investigated as an effect modifier, T stage would need to be consistently evaluated in routine clinical practice at the point of metastatic disease diagnosis. For patients with low-volume, metachronous disease, other adjuncts to ADT, such as a second-generation anti-androgen therapy might be preferable, and for those with synchronous low-volume disease, radiation to the prostate might be a less toxic, life-prolonging option.

Until other reliable biomarkers or risk groups can be identified, clinical indicators will guide most treatment choices for patients with metastatic, hormone-sensitive prostate cancer. Thus, current and future clinical trials should aim to prospectively capture reliable data on timing of metastatic disease diagnosis; volume, location and number of metastases; T stage; and biological predictors of outcome. As access to more sensitive monitoring tools such as PSMA-PET and whole-body MRI increases, less intensive treatment options, such as short-term ADT combined with stereotactic ablative radiotherapy are likely to become more prevalent in trials and in practice for low-volume disease with good prognosis. Conversely, for patients who present with poorer prognosis, high-volume metastatic disease, trials are likely to continue to focus on more intensive therapies. This analysis might guide studies to identify underlying biological reasons why some patients benefit more or less from docetaxel and will inform further research to determine which, if any, groups benefit from adding docetaxel to testosterone suppression plus a potent androgen receptor signalling inhibitor (eg, abiraterone, enzalutamide, darolutamide, and apalutamide).

The addition of docetaxel to hormone therapy is best suited to patients with poorer prognosis metastatic, hormone-sensitive prostate cancer based on a high-volume of disease and potentially the bulkiness of the primary tumour. There is no evidence of a meaningful benefit for patients with metachronous, low-volume disease, who should therefore be managed differently.

## Supplementary Material

Supplementary appendix

## Figures and Tables

**Table 1:** Trial design details and key participant characteristics

	GETUG-AFU15^5^	CHAARTED^2^	STAMPEDE^7^
Accrual period	October, 2004, to December, 2008	July, 2006, to November, 2012	November, 2005, to March, 2013
Number of patients randomly assigned	385	790	1086
Control group treatment	ADT (LHRH agonist or LHRH agonist plus anti-androgen therapy or surgical castration)	ADT (LHRH agonist or LHRH antagonist or surgical castration); oral calcium carbonate 500 mg daily; oral vitamin D 400 IU daily	ADT (GRH agonists or antagonists or orchidectomy)
Intervention group treatment	ADT (LHRH agonist or LHRH agonist plus antiandrogen therapy or surgical castration) plus docetaxel (75 mg/m^2^ intravenously every 3 weeks for a maximum of nine cycles); premedication with an oral corticosteroid (8 mg dexamethasone or equivalent) the evening before, on the day of, and on the day after docetaxel infusion plus subcutaneous injection of G-CSF from day 5 for 5 days	ADT (LHRH agonist or LHRH antagonist or surgical castration) plus docetaxel (75 mg/m^2^ intravenously every 3 weeks for six cycles); oral dexamethasone (8 mg approximately 12 h, 3 h, and 1 h before docetaxel); oral diphenhydramine optional; 500 mg oral calcium carbonate once daily; 400 IU oral vitamin D once daily	ADT (GRH agonists or antagonists or orchidectomy) plus docetaxel (75 mg/m^2^ intravenously every 3 weeks for six cycles) plus oral prednisolone (10 mg once daily)
Median follow-up for all participants (IQR), months*	84 (79–89)	54 (42–67)	78 (63–96)

ADT=androgen deprivation therapy. G-CSF=granulocyte-colony stimulating factor. GRH=gonadotropin-releasing hormone. LHRH=luteinising hormone-releasing hormone.

* Data supplied for inclusion in the meta-analysis, and follow-up duration for each trial is in keeping with the most recent version of reported trial analysis, as cited.

**Table 2:** Patient baseline characteristics

	GETUG-AFU15^5^			CHAARTED^2^			STAMPEDE^7^	
	ADT alone group (n=193)	ADT plus docetaxel group (n=192)		ADT alone group (n=393)	ADT plus docetaxel group (n=397)		ADT alone group (n=724)	ADT plus docetaxel group (n=362)
Age, years	64·3 (58·3–70·1)	63·1 (57·7–68·9)		63·0 (56·0–69·0)	64·0 (57·0–69·0)		65·9 (60·5–71·1)	65·4 (61·0–70·9)
WHO performance status								
0	176 (91%)	181 (94%)		272 (69%)	277 (70%)		521 (72%)	270 (75%)
1–2	7 (4%)	2 (1%)		121 (31%)	120 (30%)		203 (28%)	92 (25%)
Missing	10 (5%)	9 (5%)		0	0		0	0
Alkaline phosphatase, IU/L*	359 (136–570)	280 (155–545)		··	··		110 (75–253)	106 (72–233)
Missing	122 (63%)	113 (59%)		393 (100%)	397 (100%)		10 (1%)	4 (1%)
Prostate-specific antigen, ng/mL	25·9 (4·9–127·0)	26·7 (5·0–109·3)		13·7 (1·8–71·4)	10·8 (2·0–66·0)		102·5 (32·8–354·0)	97·1 (40·5–340·0)
Missing	3 (2%)	1 (1%)		1 (<1%)	0		0	0
Risk group								
High	70 (36%)	72 (38%)		169 (43%)	182 (46%)		257 (36%)	122 (34%)
Low	89 (46%)	88 (46%)		91 (23%)	105 (26%)		237 (33%)	115 (32%)
Missing	34 (18%)	32 (17%)		133 (34%)	110 (28%)		230 (32%)	125 (35%)
Gleason sum score								
<8	78 (40%)	84 (44%)		104 (26%)	117 (29%)		158 (22%)	66 (18%)
≥8	113 (59%)	103 (54%)		243 (62%)	241 (61%)		479 (66%)	253 (70%)
Missing	2 (1%)	5 (3%)		46 (12%)	39 (10%)		87 (12%)	43 (12%)
Nodal involvement								
NO	35 (18%)	31 (16%)		103 (26%)	127 (32%)		242 (33%)	118 (33%)
N+	43 (22%)	29 (15%)		138 (35%)	124 (31%)		416 (57%)	211 (58%)
Missing	115 (60%)	132 (69%)		152 (39%)	146 (37%)		66 (9%)	33 (9%)
Clinical T stage								
T1–2	45 (23%)	32 (17%)		190 (48%)	207 (52%)		90 (12%)†	52 (14%)†
T3	58 (30%)	48 (25%)		68 (17%)	63 (16%)		404 (56%)	197 (54%)
T4	3 (2%)	8 (4%)		38 (10%)	41 (10%)		163 (23%)	82 (23%)
Missing	87 (45%)	104 (54%)		97 (25%)	86 (22%)		67 (9%)	31 (9%)
Timing of metastatic disease diagnosis								
Synchronous	144 (75%)	128 (67%)		286 (73%)	289 (73%)		689 (95%)	347 (96%)
Metachronous	46 (24%)	62 (32%)		106 (27%)	108 (27%)		35 (5%)	15 (4%)
Missing	3 (2%)	2 (1%)		1 (<1%)	0		0	0
Evidence of bone metastases								
Yes	156 (81%)	157 (82%)		236 (60%)	252 (63%)		634 (88%)	307 (85%)
No	35 (18%)	34 (18%)		14 (4%)	11 (3%)		90 (12%)	55 (15%)
Missing	2 (1%)	1 (1%)		143 (36%)‡	134 (34%)‡		0	0
Evidence of visceral metastases§								
Yes	26 (13%)	31 (16%)		72 (18%)	66 (17%)		87 (12%)	40 (11%)
No	167 (87%)	161 (84%)		320 (81%)	331 (83%)		637 (88%)	322 (89%)
Missing	0	0		1 (<1%)	0		0	0
Disease volume								
Low	88 (46%)	87 (45%)		143 (36%)	134 (34%)		238 (33%)	124 (34%)
High	97 (50%)	100 (52%)		250 (64%)	263 (66%)		320 (44%)	148 (41%)
Missing	8 (4%)	5 (3%)		0	0		166 (23%)	90 (25%)

Data are median (IQR) or n (%). Percentages might not sum to 100 as a result of rounding. Data supplied for inclusion in the meta-analysis are in keeping with the most recent version of reported trial analysis, as cited. Comparison of baseline characteristics of patients by availability of clinical T stage data is in the appendix (p 51).

* In GETUG-AFU15, specific values were only recorded for patients who were noted as having values outside the normal range at randomisation; 219 participants whose baseline reading was recorded as within the normal range had no specific value noted and are included here as missing.

† Four patients randomly assigned in STAMPEDE reported as T stage 0, M stage 1 were included alongside the patients with T stage 1–2 for reporting and analysis purposes.

‡ 277 patients in CHAARTED were recorded as low volume without specifically characterising presence or absence of bone metastases.

§ Visceral metastases located in lung, liver, or adrenal gland.

**Table 3:** Absolute effects of docetaxel on 5-year progression-free survival and overall survival by volume and timing, and volume and clinical T stage combined

	Progression-free survival					Overall survival			
	Number of events/ patients*	Difference in survival at 5 years (95% CI)	5-year survival (95% CI), ADT alone	5-year survival (95% CI), ADT plus docetaxel		Number of Events / patients	Absolute effect at 5 years (95% CI)	5-year survival (95% CI), ADT alone	5-year survival (95% CI), ADT plus docetaxel
**Disease volume and timing of diagnosis**									
Low volume, metachronous†	107/229	–1% (–15 to 12)	48% (39 to 60)	47% (36 to 61)		70/229	0% (–10 to 12)	72% (63 to 82)	73% (63 to 83)
Low volume, synchronous	346/582	8% (1 to 15)	37% (33 to 42)	45% (40 to 52)		267/582	8% (0 to 16)	52% (47 to 57)	60% (54 to 66)
High volume, metachronous	92/132	11% (–2 to 24)	14% (7 to 26)	25% (15 to 41)		78/132	10% (–6 to 26)	28% (18 to 43)	38% (25 to 57)
High volume, synchronous	856/1044	12% (7 to 16)	12% (10 to 14)	23% (20 to 28)		736/1044	12% (7 to 18)	26% (23 to 30)	39% (34 to 43)
**Disease volume and clinical T stage**									
Low volume, T stage 1–3	302/569	5% (–2 to 12)	42% (37 to 47)	46% (41 to 52)		225/569	4% (–3 to 11)	58% (53 to 63)	62% (57 to 68)
Low volume, T stage 4	61/85	12% (–6 to 29)	25% (15 to 39)	36% (22 to 59)		51/85	16% (–3 to 36)	38% (26 to 54)	54% (39 to 74)
High volume, T stage 1–3	562/709	8% (4 to 13)	14% (12 to 17)	22% (18 to 26)		484/709	6% (0 to 12)	29% (25 to 32)	35% (31 to 37)
High volume, T stage 4‡	157/192	27% (17 to 37)	9% (5 to 15)	35% (27 to 47)		136/192	35% (24 to 47)	20% (14 to 29)	55% (47 to 66)

* Numbers of patients and events per subgroup as defined; however, estimates in the analysis are from a one-stage model with adjustment and imputation including all patients and events, not restricted to those shown within each subgrouping.

† 161 (70%) of 229 patients had T stage 1–3, four (2%) had T stage 4, and 64 (28%) had missing data.

‡ 188 (98%) of 192 patients had synchronous and four (2%) had metachronous.

## Data Availability

Individual participant data for GETUG-AFU15 and STAMPEDE are available from the trial investigators and sponsors through the corresponding author (CVL), subject to appropriate data sharing agreements. Data for the CHAARTED study is available on request to the NCTN/NCORP Data Archive. For the **NCTN/NCORP Data Archive** see https://nctn-data-archive.nci.nih.gov/

## Research in context

### Evidence before this study

To identify randomised trials that assessed addition of docetaxel to standard androgen deprivation therapy for patients with metastatic hormone-sensitive prostate cancer, we used highly sensitive search strategies for MEDLINE, Embase, and the Cochrane Central Register of Controlled Trials; and relevant search terms (including “metastatic hormone-sensitive prostate cancer”, “chemotherapy”, and “docetaxel”) for ClinicalTrials.gov and proceedings and abstracts of relevant conferences. Five eligible trials were identified, two of which are unpublished, with the remaining three representing an estimated 98% of eligible participants and each considered as being at low risk of bias. Results of these trials, and meta-analyses using aggregate data from them show a survival benefit of adding docetaxel to androgen depravation therapy in people with metastatic, hormone-sensitive prostate cancer. However, there was inconsistency among the trials results, about precisely which patients benefit more or less from docetaxel.

### Added value of this study

Through the collection, meticulous checking, and rigorous analysis of updated individual participant data, we have been able to add substantial value to previous evidence. We have generated updated and more precise relative and absolute estimates of the overall benefits of docetaxel. We have also appropriately investigated interactions between treatment effects and patient characteristics, accounting for other effect modifiers and prognostic factors, to corroborate associations between the effects of docetaxel, volume of metastases, and timing of metastatic disease diagnosis and identified clinical T stage as an additional potentially important effect modifier.

### Implications of all the available evidence

Our results show that the addition of docetaxel to hormone therapy is best suited to patients with poorer prognosis metastatic, hormone-sensitive prostate cancer based on a synchronous diagnosis of high-volume of disease and potentially the bulkiness of the primary tumour. Patients with metachronous, low-volume disease gain no meaningful benefit and should be managed differently. By improving the characterisation of patients most and, importantly, least likely to gain benefit from docetaxel, these results will potentially change international practice, guide clinical decision making, inform treatment policy, and improve patient outcomes. They will also inform research to identify which patients could benefit from adding docetaxel to testosterone suppression plus a potent androgen receptor signalling inhibitor.
