# Supplementary material for: Which patients with metastatic hormone-sensitive prostate cancer benefit from docetaxel: a systematic review and meta-analysis of individual participant data from randomised trials
Source: Lancet Oncol. Author manuscript; Available in PMC 2024 Aug 8. (PMC7616350; doi:10.1016/S1470-2045(23)00230-9)
Supplement: Supplementary appendix [file EMS197912-supplement-Supplementary_appendix.pdf]

Supplementary Appendix

Search Strategies *2*

MEDLINE (Cochrane Highly Sensitive Search Strategy for identifying RCTs in MEDLINE: sensitivity and precision maximising version (2008 revision)(28); Ovid format):........ 2

EMBASE Search strategy (Best Optimisation of Sensitivity and Specificity Search(29); Ovid format): 4

LILACS Search strategy: Search Strategy 1 (Highly Sensitive Search Strategy(30); Lilacs format):..... 5

Risk of Bias assessment Tables Table S1a: Summary of risk of bias assessments for all trials: Overall survival *7*

Table S1b: Summary of risk of bias assessments for all trials: Progression Free survival 18

Table S1c: Summary of risk of bias assessments for all trials: Failure Free Survival 32

Supplementary Tables........ *47*

Table S2: Sensitivity analyses: results for OS PFS and FFS........ 47

Table S3: Relative and absolute effects of docetaxel on sensitivity outcomes 48

Table S4: Effect of docetaxel on PFS by other participant characteristics 49

Table S5. Mutually-adjusted two-way interactions between volume, timing of metastases and clinical T-stage treatment 50

Table S6. Exploratory analysis showing the effect of docetaxel on PFS 51

Table S7. Comparison of baseline characteristics of patients by availability of cT stage data........ 51

Supplementary Figures *52*

Figure S1: Prisma flow diagram 52

Figure S2: Risk of Bias summary of assessments (traffic light) 53

Figure S3: Assessment of proportional hazards for (a) OS, (b) PFS and (c) FFS 54

Figure S4: Overall effects of Docetaxel for OS, PFS FFS 55

Figure S5: Absolute effects -Forest plots for OS, PFS FFS 56

Figure S6: Flow diagram of progression free survival events 57

Figure S7: Flow diagram of failure free survival events........ 58

.... 58

Figure S8: Effect of docetaxel on PFS and OS for patients with high volume disease, by timing of metastatic disease diagnosis 59

Figure S9: Effect of docetaxel on PFS and OS for patients with low volume disease, by clinical T stage........ 60

Search Strategies

MEDLINE (Cochrane Highly Sensitive Search Strategy for identifying RCTs in MEDLINE: sensitivity and precision maximising version (2008 revision)(28); Ovid format):

RCT filter MEDLINE
1. "randomi*ed controlled trial".pt.
2. controlled clinical trial.pt.
3. "randomi*ed".ab.
4. placebo.ab.
5. clinical trials as topic.sh.
6. randomly.ab.
7. trial.ti.
8. 1 or 2 or 3 or 4 or 5 or 6 or 7 or 8
9. exp animals/ not humans.sh.
10. 8 not 9

AND Terms specific to prostate cancer:

11. exp Prostatic Neoplasms/
12. (prostat$ adj3 adeno$).mp.
13. (prostat$ adj3 malignan$).mp.
14. (prostat$ adj3 canc$).mp.
15. (prostat$ adj3 carcinoma$).mp.
16. (prostat$ adj3 tumo?r$).mp.
17. (prostat$ adj3 neoplas$).mp.
18. 11 or 12 or 13 or 14 or 15 or 16 or 17

AND Terms specific to Androgen deprivation Therapy (ADT)

19. ((androgen$ or hormon$) adj3 (ablat$ or block$ or withdraw$ or depriv$ or suppress$)).mp.
20. exp Antineoplastic Agents, Hormonal/tu [Therapeutic Use]
21. exp Androgen Antagonists/
22. (luteinizing hormone releasing hormone or LHRH).mp.
23. Orchiectomy.mp.
24. 19 or 20 or 21 or 22 or 23

AND Terms specific to drug therapy:

25. exp Drug Therapy/
26. (multimodal$ or adjuvant or adjunct$).mp.
27. (together or plus or concurrent or combin$ or add$ or conjuct$).tw.
28. 25 or 26 or 27

AND Terms specific to chemotherapy

29. ((chemotherapy or antineoplastic or anticancer) adj3 (agent$).tw.
30. exp chemotherapy adjuvant/
31. exp Antineoplastic agents/
32. docetaxel.tw.
33. taxotere.tw.
34. 29 or 30 or 31 or 32 or 33

AND Terms specific to bisphosphonates:

35. exp diphosphonates/
36. exp bisphosphonates/
37. (bisphosphonate$ or disphosphonat$).af.
38. zoledron$.af.
39. zometa.af.
40. aclasta.af.
41. 35 or 36 or 37 or 38 or 39 or 40

COMBINE ALL

42. 10 and 18 and 24 and 28
43. 34 or 41
44. 42 and 43

/ means all subheadings were selected

pt = publication type
mp = free text search for a term
tw term in a title/ abstract
af term in all fields

EMBASE Search strategy (Best Optimisation of Sensitivity and Specificity Search(29); Ovid format):

RCT filter EMBASE
1. randomi:.tw.
2. placebo:.mp.
3. double-blind:.tw.
4. 1 or 2 or 3

AND Terms specific to prostate cancer:
5. exp Prostatic Neoplasms/
6. (prostat$ adj3 adeno$).mp.
7. (prostat$ adj3 malignan$).mp.
8. (prostat$ adj3 canc$).mp.
9. (prostat$ adj3 carcinoma$).mp.
10. (prostat$ adj3 tumo?r$).mp.
11. (prostat$ adj3 neoplas$).mp.
12. 5 or 6 or 7 or 8 or 9 or 10 or 11

AND Terms specific to Androgen deprivation Therapy (ADT)
13. ((androgen$ or hormon$) adj3 (ablat$ or block$ or withdraw$ or depriv$ or suppress$)).mp.
14. exp Antineoplastic Agents, Hormonal/tu [Therapeutic Use]
15. exp Androgen Antagonists/
16. (luteinizing hormone releasing hormone or LHRH).mp.
17. Orchi$ectomy.mp.
18. 13 or 14 or 15 or 16 or 17

AND Terms specific to drug therapy:
19. exp Drug Therapy/
20. (multimodal$ or adjuvant or adjunct$).mp.
21. (together or plus or concurrent or combin$ or add$ or conjuct$).tw.
22. 19 or 20 or 21

AND Terms specific to chemotherapy
23. ((chemotherapy or antineoplastic or anticancer) adj3 (agent$).tw.
24. exp chemotherapy adjuvant/
25. exp Antineoplastic agents/
26. docetaxel.tw.
27. taxotere.tw.
28. 23 or 24 or 25 or 26 or 27

AND Terms specific to bisphosphonates:
29. exp diphosphonates/
30. exp bisphosphonates/
31. (bisphosphonate$ or disphosphonat$).af.
32. zoledron$.af.
33. zometa.af.
34. aclasta.af.
35. 29 or 30 or 31 or 32 or 33 or 34

COMBINE ALL
36. 4 and 12 and 18 and 22
37. 28 or 35
38. 36 and 37

LILACS Search strategy: Search Strategy 1 (Highly Sensitive Search Strategy(30); Lilacs format):

(Tw estud$ OR Tw clinic$ OR AB grupo$ OR CT COMPARATIVE STUDY OR Tw placebo$ OR Tw random$ OR Ti compara$ OR Ti tratamiento OR Tw control$ OR MH/dt) AND NOT ((CT ANIMALS FEMALE OR CT ANIMALS MALE OR CT CATS OR CT CATTLE OR CT CHICK EMBRYO OR CT DOGS OR CT GUINEA PIGS OR CT IN VITRO OR CT MICE OR CT RABBITS OR CT RATS) OR (MH Prevalence OR MH Practice Guidelines OR MH Diagnosis, Differential OR MH Cross-Sectional Studies OR MH predictive value of tests) OR (Ti clinical AND case OR Ti updat$ OR Ti Epidemiol$ OR Ti clinical$ AND case$ OR Ti caso AND clinico OR Ti review OR Ti diagno$ AND treatment OR Ti descrip$ OR Ti consenso OR Ti caso$ AND control$ OR Ti analisis AND critico) OR (AB retrospectiv$ and stud$ OR AB estudio AND retrospectivo OR AB revis$ AND ficha$ OR AB revision AND bibliograf$ OR AB estud$ AND descript$ OR AB presenta AND caso OR AB describe AND caso OR AB serie AND clinica OR AB puesta AND al AND dia OR AB tratamiento AND diagnostic$ AND revis$ OR AB experien$ AND caso$ OR AB analisis AND critico) OR (PT case reports OR PT review) AND NOT (Tw estud$ OR AB grupo$ OR Tw control$ OR Tw random$)) [Words]
And
(Mh prostatic neoplasms/) or (tw prostat$ AND (Tw carcinoma$ or Tw canc$ or Tw tumo$ or Tw neoplas$)) [Words]
And
(Mh Drug therapy/) or (Tw chemotherapy) [Words]
(Mh prostatic neoplasms/) or (tw prostat$ AND (Tw carcinoma$ or Tw canc$ or Tw tumo$ or Tw neoplas$)) And (Mh Drug therapy/) or (Tw chemotherapy)

Search Strategy 2: Sensitive search strategy to retrieve CLINICAL TRIALS in the LILACS database (From Bireme slide presentation: Search for information and scientific evidence at LILACS BIREME/PAHO/WHO CRICS9-BVS6 (October 22-23th, 2012)
http://programa.crics9.org/es/files/2012/08/RoseLILACS_CRICS.pdf)

((**(**PT:"Ensaio Clinico Controlado Aleatorio" OR PT:"Ensaio Clinico Controlado" OR PT:"Ensaio Clinico" OR MH:"Ensaios Clinicos Controlados Aleatorios como Assunto" OR MH:"Ensaios Clinicos Controlados como Assunto" OR MH:"Ensaios Clinicos como Assunto" OR PT:"Estudo Multicentrico" OR MH:"Distribuicao Aleatoria" OR MH:"Metodo Duplo-Cego" OR MH:"Metodo Simples-Cego"**)** OR ((MH:"Grupos Controle" OR MH:"Estudos Cross-Over" OR MH:"Estudos de seguimento" or MH:"Estudos prospectivos" OR PT:"Estudo comparativo" OR PT:"Estudos de avaliacao" OR MH:"Projetos de Pesquisa") AND ((tw:ensaio$ or tw:ensayo$ or tw:trial$ or tw:estudo$ or tw:estudio$ or tw:study or tw:studies) AND (tw:azar or tw:acaso or tw:enmascarado or tw:placebo$ or tw:control$ or tw:aleat$ or tw:random$ or tw:dobleciego or tw:simpleciego or ((tw:simple$ or tw:single or tw:duplo$ or tw:doble$ or tw:double$ or tw:trebl$ or tw:tripl$) and (tw:cego$ or tw:ciego$ or tw:blind$ or tw:mask$))) AND tw:clinic$)) OR ((tw:ensaio$ or tw:ensayo$ or tw:trial$) AND (tw:azar or tw:acaso or tw:enmascarado or tw:placebo$ or tw:control$ or tw:aleat$ or tw:random$ or tw:dobleciego or tw:simpleciego or ((tw:simple$ or tw:single$ or tw:duplo$ or tw:doble$ or tw:double$ or tw:trebl$ or tw:tripl$) and (tw:cego or tw:ciego or tw:blind or tw:mask))) AND tw:clinic$)) AND NOT (MH:animais OR MH:ratos OR MH:camundongoS OR MH:gatos OR MH:primatas OR MH:caes OR MH:coelhos OR MH:suinos OR PT:"in vitro"))

- **Controlled Clinical Trials / Ensaios Clínicos Controlados / Ensayos Clínicos Controlados**

((PT:"randomized controlled trial**" OR PT:"**controlled clinical trial**" OR PT:"**multicenter study**" OR MH:"**randomized controlled trials as topic**" OR MH:"**controlled clinical trials as topic**" OR MH:"**multicenter studies as topic**" OR MH:"**random allocation**" OR MH:"**double-blind method" **OR MH:"**single-blind method"**) OR ((**ensaio**$ OR** ensayo**$ OR** trial**$) AND (**azar **OR** acaso **OR** placebo **OR** control**$ OR** aleat**$ OR** random**$ OR** enmascarado**$ OR** simpleciego **OR ((**simple**$ OR** single **OR** duplo**$ OR** doble**$ OR** double**$) AND (**cego **OR** ciego **OR** blind **OR** mask**))) AND** clinic**$)) AND NOT (MH:**animals **OR MH:**rabbits **OR MH:**rats **OR MH:**primates **OR MH:**dogs **OR MH:**cats **OR MH:**swine **OR PT:"**in vitro**")**
(MH:prostatic neoplasms) or (tw prostat$ AND (Tw carcinoma$ or Tw canc$ or Tw tumo$ or Tw neoplas$)) And (Mh Drug therapy/) or (Tw chemotherapy)

Search Strategy 3 Ensaios clínicos randomizados (From Brazilian Cochrane Center website, March 2014)

((Pt randomized controlled trial OR Pt controlled clinical trial OR Mh randomized controlled trials OR Mh random allocation OR Mh double-blind method OR Mh single-blind method) AND NOT (Ct animal AND NOT (Ct human and Ct animal)) OR (Pt clinical trial OR Ex E05.318.760.535$ OR (Tw clin$ AND (Tw trial$ OR Tw ensa$ OR Tw estud$ OR Tw experim$ OR Tw investiga$)) OR ((Tw singl$ OR Tw simple$ OR Tw doubl$ OR Tw doble$ OR Tw duplo$ OR Tw trebl$ OR Tw trip$) AND (Tw blind$ OR Tw cego$ OR Tw ciego$ OR Tw mask$ OR Tw mascar$)) OR Mh placebos OR Tw placebo$ OR (Tw random$ OR Tw randon$ OR Tw casual$ OR Tw acaso$ OR Tw azar OR Tw aleator$) OR Mh research design) AND NOT (Ct animal AND NOT (Ct human and Ct animal)) OR (Ct comparative study OR Ex E05.337$ OR Mh follow-up studies OR Mh prospective studies OR Tw control$ OR Tw prospectiv$ OR Tw volunt$ OR Tw volunteer$) AND NOT (Ct animal AND NOT (Ct human and Ct animal)))

(MH:prostatic neoplasms) or (tw prostat$ AND (Tw carcinoma$ or Tw canc$ or Tw tumo$ or Tw neoplas$)) And (Mh Drug therapy/) or (Tw chemotherapy)

Risk of Bias assessment Tables

Table S1a: Summary of risk of bias assessments for all trials: Overall survival

**Study ID**	**STAMPEDE A vs C (OS)**		
**Domain**	**Signalling question**	**Response**	**Comments**
**Bias arising from the randomization process**	1.1 Was the allocation sequence random?	Y	(Based on the manuscript) The allocation sequence was generated by minimisation with a random element of 80% stratifying for hospital, age at randomisation, presence of metastases, planned radiotherapy use, nodal involvement, WHO performance status, planned hormone therapy, and regular use of aspirin or another non-steroidal anti-inflammatory drug. NB: Allocation was in a 2:1 ratio to standard of care only (SOC-only), standard of care plus docetaxel (SOC + DOC).<br><br>(Based on direct checks of the IPD) The cumulative number of participants allocated to each intervention group (accounting for 2:1 allocation ratio) was balanced over time; participants were allocated to a similar degree to each intervention group on each day of the week and there were no weekend randomisations (which is the usual for cancer trials).
	1.2 Was the allocation sequence concealed until participants were enrolled and assigned to interventions?		(Based on the manuscript) The allocation sequence was concealed as participants were allocated centrally using a computerised algorithm developed and maintained by the MRC CTU.
	1.3 Did baseline differences between intervention groups suggest a problem with the randomization process?	N	(Based on direct checks of the IPD) The baseline characteristics age, performance status, disease stage, Gleason score, location of metastases, volume of disease, risk status, alkaline phosphatase, PSA, and BMI were well balanced by intervention group.

	**Domain 1: Risk of bias judgement**	**Low**	
**Bias due to deviations from intended interventions**	2.1.Were participants aware of their assigned intervention during the trial?	Y	As this was a trial of docetaxel chemotherapy plus hormone therapy versus hormone therapy, with different modes of administration and side effects, participants were necessarily aware of the assigned intervention.
	2.2.Were carers and people delivering the interventions aware of participants' assigned intervention during the trial?		As this was a trial of docetaxel chemotherapy plus hormone therapy versus hormone therapy, with different modes of administration and side effects, carers and those delivering interventions were necessarily aware of the assigned intervention.
	2.3. If Y/PY/NI to 2.1 or 2.2: Were there deviations from the intended intervention that arose because of the experimental context?	N	We are not aware of any deviations because of the trial context.
	2.4 If Y/PY to 2.3: Were these deviations likely to have affected the outcome?	NA	
	2.5. If Y/PY/NI to 2.4: Were these deviations from intended intervention balanced between groups?	NA	
	2.6 Was an appropriate analysis used to estimate the effect of assignment to intervention?	Y	Using the IPD, we conducted an appropriate intention-to-treat analysis of all randomised participants.
	2.7 If N/PN/NI to 2.6: Was there potential for a substantial impact (on the result) of the failure to analyse participants in the group to which they were randomized?	NA	
	**Domain 2: Risk of bias judgement**	**Low**	
	3.1 Were data for this outcome available for all, or nearly all, participants randomized?	Y	IPD for overall survival were available for all participants randomised.

**Bias due to missing outcome data**	3.2 If N/PN/NI to 3.1: Is there evidence that result was not biased by missing outcome data?	NA	
	3.3 If N/PN to 3.2: Could missingness in the outcome depend on its true value?	NA	
	3.4 If Y/PY/NI to 3.3: Is it likely that missingness in the outcome depended on its true value?		
	**Domain 3: Risk of bias judgement**	**Low**	
**Bias in measurement of the outcome**	4.1 Was the method of measuring the outcome inappropriate?	N	Overall survival is an objective and appropriate outcome. It was derived from the supplied IPD according to a standardised meta-analysis definition.
	4.2 Could measurement or ascertainment of the outcome have differed between intervention groups?	N	(Based on direct checks of the IPD) The duration of follow-up is sufficient (median = 6.5 years) and balanced across intervention groups.
	4.3 Were outcome assessors aware of the intervention received by study participants?	Y	Yes
	4.4 If Y/PY/NI to 4.3: Could assessment of the outcome have been influenced by knowledge of intervention received?	N	Assessment of overall survival could not be influenced by knowledge of the intervention.
	4.5 If Y/PY/NI to 4.4: Is it likely that assessment of the outcome was influenced by knowledge of intervention received?	NA	
	**Domain 4: Risk of bias judgement**	**Low**	
**Bias in selection of the reported result**	5.1 Were the data that produced this result analysed in accordance with a pre-specified analysis plan that was finalized before	Y	Our analysis of the trial and the meta-analysis are derived directly from the IPD and not based on reported results. Both follow a statistical analysis plan, specified before the analysis was conducted, unless otherwise stated.

	unblinded outcome data were available for analysis?		
	5.2 ... multiple eligible outcome measurements (e.g. scales, definitions, time points) within the outcome domain?	N	
	5.3 ... multiple eligible analyses of the data?	N	
	**Domain 5: Risk of bias judgement**	**Low**	
**Overall bias**	**Risk of bias judgement: STAMPEDE A vs C (OS)**	**Low**	

**Study ID**	**GETUG-15 (OS)**		
**Domain**	**Signalling question**	**Response**	**Comments**
**Bias arising from the randomization process**	1.1 Was the allocation sequence random?	Y	(Based on the manuscript) Patients were randomly allocated in a 1:1 ratio to receive ADT plus docetaxel or ADT alone. Dynamic minimisation was used to minimise the imbalance of three criteria: previous systemic treatment with ADT; chemotherapy for local disease or isolated rising PSA; and Glass risk groups<br><br>(Based on direct checks of the IPD) The cumulative number of participants allocated to each intervention group is balanced over time; participants were allocated to a similar degree to each intervention group on each day of the week and there were no weekend randomisations (which is the usual for cancer trials).
	1.2 Was the allocation sequence concealed until participants were enrolled and assigned to interventions?		

			(Based on the manuscript) Treatment allocation was done by a clinical research organisation and was centralised nationally.
	1.3 Did baseline differences between intervention groups suggest a problem with the randomization process?	N	(Based on direct checks of the IPD) The baseline characteristics age, performance status, disease stage, method of diagnosis, Gleason score, location of metastases, volume of disease, risk status, alkaline phosphatase, number of bone metastases, PSA, and BMI were well balanced by intervention group.
	**Domain 1: Risk of bias judgement**	**Low**	
**Bias due to deviations from intended interventions**	2.1.Were participants aware of their assigned intervention during the trial?	Y	As this was a trial of docetaxel chemotherapy plus hormone therapy versus hormone therapy, with different modes of administration and side effects, participants were necessarily aware of the assigned intervention
	2.2.Were carers and people delivering the interventions aware of participants' assigned intervention during the trial?		As this was a trial of docetaxel chemotherapy plus hormone therapy versus hormone therapy, with different modes of administration and side effects, carers and those delivering interventions were necessarily aware of the assigned intervention.
	2.3. If Y/PY/NI to 2.1 or 2.2: Were there deviations from the intended intervention that arose because of the experimental context?	N	We are not aware of any deviations because of the trial context
	2.4 If Y/PY to 2.3: Were these deviations likely to have affected the outcome?	NA	
	2.5. If Y/PY/NI to 2.4: Were these deviations from intended intervention balanced between groups?	NA	
	2.6 Was an appropriate analysis used to estimate the effect of assignment to intervention?	Y	Using the IPD, we conducted an appropriate intention-to-treat analysis of all randomised participants

	2.7 If N/PN/NI to 2.6: Was there potential for a substantial impact (on the result) of the failure to analyse participants in the group to which they were randomized?	NA	
	**Domain 2: Risk of bias judgement**	**Low**	
**Bias due to missing outcome data**	3.1 Were data for this outcome available for all, or nearly all, participants randomized?	Y	IPD for overall survival were available for all participants randomised
	3.2 If N/PN/NI to 3.1: Is there evidence that result was not biased by missing outcome data?	NA	
	3.3 If N/PN to 3.2: Could missingness in the outcome depend on its true value?	NA	
	3.4 If Y/PY/NI to 3.3: Is it likely that missingness in the outcome depended on its true value?		
	**Domain 3: Risk of bias judgement**	**Low**	
**Bias in measurement of the outcome**	4.1 Was the method of measuring the outcome inappropriate?	N	Overall survival is an objective and appropriate outcome. It was derived from the supplied IPD according to a standardised meta-analysis definition.
	4.2 Could measurement or ascertainment of the outcome have differed between intervention groups?	N	(Based on direct checks of the IPD) The duration of follow-up is sufficient (median = 7 years) and balanced across intervention groups.
	4.3 Were outcome assessors aware of the intervention received by study participants?	Y	Yes
	4.4 If Y/PY/NI to 4.3: Could assessment of the outcome have been influenced by knowledge of intervention received?	PN	Assessment of overall survival could not be influenced by knowledge of the intervention

	4.5 If Y/PY/NI to 4.4: Is it likely that assessment of the outcome was influenced by knowledge of intervention received?	NA	
	**Domain 4: Risk of bias judgement**	**Low**	
**Bias in selection of the reported result**	5.1 Were the data that produced this result analysed in accordance with a pre-specified analysis plan that was finalized before unblinded outcome data were available for analysis?	Y	Our analysis of the trial and the meta-analysis are derived directly from the IPD and not based on reported results. Both follow a statistical analysis plan, specified before the analysis was conducted, unless otherwise stated
	5.2 ... multiple eligible outcome measurements (e.g. scales, definitions, time points) within the outcome domain?	N	
	5.3 ... multiple eligible analyses of the data?	N	
	**Domain 5: Risk of bias judgement**	**Low**	
**Overall bias**	**Risk of bias judgement: GETUG-15 (OS)**	**Low**	
**Study ID**	**CHAARTED (OS)**		
**Domain**	**Signalling question**	**Response**	**Comments**
**Bias arising from the**	1.1 Was the allocation sequence random?	Y	(Based on the protocol) 'The method of permuted blocks will be used for subject randomization. No per-site treatment-allocation balance

**randomization process**	1.2 Was the allocation sequence concealed until participants were enrolled and assigned to interventions?		will be implemented'<br><br>(Based on the manuscript) Patients were stratified according to age (<70 years vs. ≥70 years), ECOG performance-status score (0 or 1 vs. 2), and planned use of combined androgen blockade for more than 30 days (yes vs. no) or agents approved for prevention of skeletal-related events in castration-resistant disease (zoledronic acid or denosumab) (yes vs. no). Patients were also stratified according to the duration of prior adjuvant ADT (<12 months vs. ≥12 months) and the extent of metastases (high volume [defined as the presence of visceral metastases or ≥4 bone lesions with ≥1 beyond the vertebral bodies and pelvis] vs. low volume).<br><br>(Based on direct checks of the IPD) The cumulative number of participants allocated to each intervention group is balanced over time; participants were allocated to a similar degree to each intervention group on each day of the week and there were no weekend randomisations (which is the usual for cancer trials).<br><br>1.2 (Based on the manuscript) The sequence was concealed as participants were allocated centrally via a Central Randomisation Desk at the ECOG coordinating centre.
	1.3 Did baseline differences between intervention groups suggest a problem with the randomization process?	N	(Based on direct checks of the IPD) The baseline characteristics age, performance status, disease stage, Gleason score, location of metastases, volume of disease, risk status, number of bone metastases, PSA and BMI were well balanced by intervention group.
	**Domain 1: Risk of bias judgement**	**Low**	
**Bias due to deviations**	2.1.Were participants aware of their assigned intervention during the trial?	Y	As this was a trial of docetaxel chemotherapy plus hormone therapy versus hormone therapy, with different modes of administration and

**from intended interventions**	2.2.Were carers and people delivering the interventions aware of participants' assigned intervention during the trial?		side effects, participants were necessarily aware of the assigned intervention.<br><br>As this was a trial of docetaxel chemotherapy plus hormone therapy versus hormone therapy, with different modes of administration and side effects, carers and those delivering interventions were necessarily aware of the assigned intervention.
	2.3. If Y/PY/NI to 2.1 or 2.2: Were there deviations from the intended intervention that arose because of the experimental context?	N	We are not aware of any deviations because of the trial context.
	2.4 If Y/PY to 2.3: Were these deviations likely to have affected the outcome?	NA	
	2.5. If Y/PY/NI to 2.4: Were these deviations from intended intervention balanced between groups?	NA	
	2.6 Was an appropriate analysis used to estimate the effect of assignment to intervention?	Y	Using the IPD, we conducted an appropriate intention-to-treat analysis of all randomised participants.
	2.7 If N/PN/NI to 2.6: Was there potential for a substantial impact (on the result) of the failure to analyse participants in the group to which they were randomized?	NA	
	**Domain 2: Risk of bias judgement**	**Low**	
**Bias due to missing outcome data**	3.1 Were data for this outcome available for all, or nearly all, participants randomized?	Y	IPD for overall survival were available for all participants randomised.
	3.2 If N/PN/NI to 3.1: Is there evidence that result was not biased by missing outcome data?	NA	
	3.3 If N/PN to 3.2: Could missingness in the outcome depend on its true value?	NA	

	3.4 If Y/PY/NI to 3.3: Is it likely that missingness in the outcome depended on its true value?		
	**Domain 3: Risk of bias judgement**	**Low**	
**Bias in measurement of the outcome**	4.1 Was the method of measuring the outcome inappropriate?	N	Overall survival was derived from the supplied IPD according to a standardised meta-analysis definition
	4.2 Could measurement or ascertainment of the outcome have differed between intervention groups?	N	(Based on direct checks of the IPD) The duration of follow-up is sufficient (median = 4.5 years) and balanced across intervention groups.
	4.3 Were outcome assessors aware of the intervention received by study participants?	Y	Yes
	4.4 If Y/PY/NI to 4.3: Could assessment of the outcome have been influenced by knowledge of intervention received?	N	Assessment of overall survival could not be influenced by knowledge of the intervention.
	4.5 If Y/PY/NI to 4.4: Is it likely that assessment of the outcome was influenced by knowledge of intervention received?	NA	
	**Domain 4: Risk of bias judgement**	**Low**	
**Bias in selection of the reported result**	5.1 Were the data that produced this result analysed in accordance with a pre-specified analysis plan that was finalized before unblinded outcome data were available for analysis?	Y	Our analysis of the trial and the meta-analysis are derived directly from the IPD and not based on reported results. Both follow a statistical analysis plan, specified before the analysis was conducted, unless otherwise stated.
	5.2 ... multiple eligible outcome measurements (e.g. scales, definitions, time points) within the outcome domain?	N	
	5.3 ... multiple eligible analyses of the data?	N	

	**Domain 5: Risk of bias judgement**	**Low**	
**Overall bias**	**Risk of bias judgement: CHAARTED (OS)**	**Low**	

Table S1b: Summary of risk of bias assessments for all trials: Progression Free survival

**Study ID**	**STAMPEDE A vs C (PFS)**		
**Domain**	**Signalling question**	**Response**	**Comments**
**Bias arising from the randomization process**	1.1 Was the allocation sequence random?	Y	(Based on the manuscript) The allocation sequence was generated by minimisation with a random element of 80% stratifying for hospital, age at randomisation, presence of metastases, planned radiotherapy use, nodal involvement, WHO performance status, planned hormone therapy, and regular use of aspirin or another non-steroidal anti-inflammatory drug. NB: Allocation was in a 2:1 ratio to standard of care only (SOC-only), standard of care plus docetaxel (SOC + DOC).<br><br>(Based on direct checks of the IPD) The cumulative number of participants allocated to each intervention group (accounting for 2:1 allocation ratio) was balanced over time; participants were allocated to a similar degree to each intervention group on each day of the week and there were no weekend randomisations (which is the usual for cancer trials).<br><br>(Based on the manuscript) The allocation sequence was concealed as participants were allocated centrally using a computerised algorithm developed and maintained by the MRC CTU.
	1.2 Was the allocation sequence concealed until participants were enrolled and assigned to interventions?		
	1.3 Did baseline differences between intervention groups suggest a problem with the randomization process?	N	(Based on direct checks of the IPD) The baseline characteristics age, performance status, disease stage, Gleason score, location of metastases, volume of disease, risk status, alkaline phosphatase, PSA, and BMI were well balanced by intervention group.
	**Risk of bias judgement**	**Low**	

**Bias due to deviations from intended interventions**	2.1.Were participants aware of their assigned intervention during the trial?	Y	As this was a trial of docetaxel chemotherapy plus hormone therapy versus hormone therapy, with different modes of administration and side effects, participants were necessarily aware of the assigned intervention.
	2.2.Were carers and people delivering the interventions aware of participants' assigned intervention during the trial?	Y	As this was a trial of docetaxel chemotherapy plus hormone therapy versus hormone therapy, with different modes of administration and side effects, carers and those delivering interventions were necessarily aware of the assigned intervention.
	2.3. If Y/PY/NI to 2.1 or 2.2: Were there deviations from the intended intervention that arose because of the experimental context?	N	We are not aware of any deviations because of the trial context.
	2.4 If Y/PY to 2.3: Were these deviations likely to have affected the outcome?	NA	
	2.5. If Y/PY/NI to 2.4: Were these deviations from intended intervention balanced between groups?	NA	
	2.6 Was an appropriate analysis used to estimate the effect of assignment to intervention?	Y	Using the IPD, we conducted an appropriate intention-to-treat analysis of all randomised participants.
	2.7 If N/PN/NI to 2.6: Was there potential for a substantial impact (on the result) of the failure to analyse participants in the group to which they were randomized?	NA	
	**Risk of bias judgement**	**Low**	
**Bias due to missing outcome data**	3.1 Were data for this outcome available for all, or nearly all, participants randomized?	Y	IPD for progression-free survival were available for all participants randomised.
	3.2 If N/PN/NI to 3.1: Is there evidence that result was not biased by missing outcome data?	NA	

	3.3 If N/PN to 3.2: Could missingness in the outcome depend on its true value?	NA	
	3.4 If Y/PY/NI to 3.3: Is it likely that missingness in the outcome depended on its true value?	NA	
	**Risk of bias judgement**	**Low**	
**Bias in measurement of the outcome**	4.1 Was the method of measuring the outcome inappropriate?	N	Progression-free survival and the events that comprise it (clinical progression, radiological progression and death) are standard and appropriate measures for assessing the effects of treatment on prostate cancer progression. The time to all these events was collected for this trial.
	4.2 Could measurement or ascertainment of the outcome have differed between intervention groups?	PN	It is a composite outcome based on clinical progression, radiological progression, or death, whichever occurred first. Measurement of the individual events by intervention group were as follows:<br><br>(Based on trial case report forms and information provided by the trialists) Clinical progression was not formally defined for this trial. Therefore, as per the meta-analysis data dictionary, clinical progression events comprise any skeletal-related event (SRE), which included bone pain, bone fracture, spinal cord compression documented for this trial.<br><br>(Based on the protocol) Participants were clinically assessed by the investigating physician at each visit: at baseline; weeks 6, 12, 18 and 24; and then every 12 weeks to 2 years; every 6 months to 5 years, then annually on both arms.<br><br>Although this outcome is somewhat subjective, awareness of the intervention is unlikely to affect assessment of such clinical symptoms.<br><br>(Based on direct checks of the IPD) Also, as expected, there is good

			correlation between the incidence of clinical and subsequent radiological progression.<br><br>(Based on protocol) Radiological progression in this trial was defined as at least a 20% increase in the sum of longest dimension (LD) target lesions taking as reference the smallest sum LD recorded since study entry and/or the appearance of one or more new lesions) and is unlikely to have been influenced by knowledge of the intervention.<br><br>(Based on the protocol and information provided by the trialists) Radiological progression was assessed for all patients at baseline, with a repeat scan advised (but not mandated) at 24 weeks or whenever deemed clinically relevant. Clinically relevant was defined by the trial team as anything that would have triggered the clinician's concern that the patient's cancer was getting worse (e.g., rising PSA, SREs or global general deterioration).<br><br>(Based on direct checks of the IPD) The duration of follow-up is balanced across intervention groups.
	4.3 Were outcome assessors aware of the intervention received by study participants?	Y	Yes
	4.4 If Y/PY/NI to 4.3: Could assessment of the outcome have been influenced by knowledge of intervention received?	PN	This is a composite outcome based on clinical progression, radiological progression, or death, whichever occurred first. It is unlikely that the events that make up the outcome could have been influenced by knowledge of intervention received given the criteria described in 4.2.<br><br>Moreover, (based on direct checks of the IPD), the more objectively measured events dominate this outcome (radiological 51% and deaths 21%) compared to the more subjective events (clinical 28%).
	4.5 If Y/PY/NI to 4.4: Is it likely that assessment of the outcome was influenced by knowledge of intervention received?	NA	

			Additionally, assessment of death could not be influenced by knowledge of the intervention.
	**Risk of bias judgement**	**Low**	
**Bias in selection of the reported result**	5.1 Were the data that produced this result analysed in accordance with a pre-specified analysis plan that was finalized before unblinded outcome data were available for analysis?	Y	Our analysis of the trial and the meta-analysis are derived directly from the IPD and not based on reported results. Both follow a statistical analysis plan, specified before the analysis was conducted, unless otherwise stated.
	5.2 ... multiple eligible outcome measurements (e.g. scales, definitions, time points) within the outcome domain?	N	
	5.3 ... multiple eligible analyses of the data?	N	
	**Risk of bias judgement**	**Low**	
**Overall bias**	**Risk of bias judgement**	**Low**	
**Study ID**	**GETUG-15 (PFS)**		
**Domain**	**Signalling question**	**Response**	**Comments**
**Bias arising from the randomization process**	1.1 Was the allocation sequence random?	Y	(Based on the manuscript) Patients were randomly allocated in a 1:1 ratio to receive ADT plus docetaxel or ADT alone. Dynamic minimisation was used to minimise the imbalance of three criteria: previous systemic treatment with ADT; chemotherapy for local disease or isolated rising PSA; and Glass risk groups<br><br>(Based on direct checks of the IPD) The cumulative number of
	1.2 Was the allocation sequence concealed until participants were enrolled and assigned to interventions?		

			participants allocated to each intervention group is balanced over time; participants were allocated to a similar degree to each intervention group on each day of the week and there were no weekend randomisations (which is the usual for cancer trials).<br><br>(Based on the manuscript) Treatment allocation was done by a clinical research organisation and was centralised nationally.
	1.3 Did baseline differences between intervention groups suggest a problem with the randomization process?	N	(Based on direct checks of the IPD) The baseline characteristics age, performance status, disease stage, method of diagnosis, Gleason score, location of metastases, volume of disease, risk status, alkaline phosphatase, number of bone metastases, PSA, and BMI were well balanced by intervention group.
	**Risk of bias judgement**	**Low**	
**Bias due to deviations from intended interventions**	2.1.Were participants aware of their assigned intervention during the trial?	Y	As this was a trial of docetaxel chemotherapy plus hormone therapy versus hormone therapy, with different modes of administration and side effects, participants were necessarily aware of the assigned intervention
	2.2.Were carers and people delivering the interventions aware of participants' assigned intervention during the trial?		As this was a trial of docetaxel chemotherapy plus hormone therapy versus hormone therapy, with different modes of administration and side effects, carers and those delivering interventions were necessarily aware of the assigned intervention.
	2.3. If Y/PY/NI to 2.1 or 2.2: Were there deviations from the intended intervention that arose because of the experimental context?	N	We are not aware of any deviations because of the trial context
	2.4 If Y/PY to 2.3: Were these deviations likely to have affected the outcome?	NA	
	2.5. If Y/PY/NI to 2.4: Were these deviations from intended intervention balanced between groups?	NA	

	2.6 Was an appropriate analysis used to estimate the effect of assignment to intervention?	Y	Using the IPD, we conducted an appropriate intention-to-treat analysis of all randomised participants
	2.7 If N/PN/NI to 2.6: Was there potential for a substantial impact (on the result) of the failure to analyse participants in the group to which they were randomized?	NA	
	**Risk of bias judgement**	**Low**	
**Bias due to missing outcome data**	3.1 Were data for this outcome available for all, or nearly all, participants randomized?	Y	IPD for progression-free survival were available for all participants randomised.
	3.2 If N/PN/NI to 3.1: Is there evidence that result was not biased by missing outcome data?	NA	
	3.3 If N/PN to 3.2: Could missingness in the outcome depend on its true value?	NA	
	3.4 If Y/PY/NI to 3.3: Is it likely that missingness in the outcome depended on its true value?		
	**Risk of bias judgement**	**Low**	
**Bias in measurement of the outcome**	4.1 Was the method of measuring the outcome inappropriate?	N	Progression-free survival and the events that comprise it (clinical progression, radiological progression, or death) are standard and appropriate measures for assessing the effects of treatment on prostate cancer progression. Although clinical (symptomatic) progression was not collected for this trial; the time to both radiological progression and death were collected.
	4.2 Could measurement or ascertainment of the outcome have differed between intervention groups?	PN	This is a composite outcome based on radiological progression or death, whichever occurred first. Measurement of the individual events by intervention group were as follows:<br><br>(Based on the protocol) Radiological progression in this trial was defined in the trial as the progression of pre-existing lesions with

			Response Evaluation Criteria in Solid Tumors (RECIST; version 1.0) or the occurrence of (new) bone lesions, whichever happened first) and is unlikely to have been influenced by knowledge of the intervention.<br><br>(Based on the protocol) Radiological progression was assessed for all patients at baseline and then every 12 weeks for up to 3.5 years; then every 6 months until progression on both arms.<br><br>(Based on direct checks of the IPD) The duration of follow-up is balanced across intervention groups.
	4.3 Were outcome assessors aware of the intervention received by study participants?	Y	Yes
	4.4 If Y/PY/NI to 4.3: Could assessment of the outcome have been influenced by knowledge of intervention received?	PN	This is a composite outcome based on radiological progression or death, whichever occurred first. It is unlikely that the events that make up the outcome could have been influenced by knowledge of intervention received given the criteria described in 4.2.<br><br>Moreover, this outcome is solely comprised of the more objective events as clinical (symptomatic) progression was not collected for this trial.<br><br>Additionally, assessment of death could not be influenced by knowledge of the intervention.
	4.5 If Y/PY/NI to 4.4: Is it likely that assessment of the outcome was influenced by knowledge of intervention received?	NA	
	**Risk of bias judgement**	**Low**	
**Bias in selection of**	5.1 Were the data that produced this result analysed in accordance with a pre-specified analysis plan that was finalized before unblinded outcome data were available for analysis?	Y	Our analysis of the trial and the meta-analysis are derived directly from the IPD and not based on reported results. Both follow a statistical analysis plan, specified before the analysis was conducted, unless otherwise stated

**the reported result**	5.2 ... multiple eligible outcome measurements (e.g. scales, definitions, time points) within the outcome domain?	N	
	5.3 ... multiple eligible analyses of the data?	N	
	**Risk of bias judgement**	**Low**	Our analysis of the trial and the meta-analysis are derived directly from the IPD and not based on reported results. Both follow a statistical analysis plan, specified before the analysis was conducted, unless otherwise stated
**Overall bias**	**Risk of bias judgement**	**Low**	
**Study ID**	**CHAARTED (PFS)**		
**Domain**	**Signalling question**	**Response**	**Comments**
**Bias arising from the randomization process**	1.1 Was the allocation sequence random?	Y	(Based on the protocol) 'The method of permuted blocks will be used for subject randomization. No per-site treatment-allocation balance will be implemented'<br><br>(Based on the manuscript) Patients were stratified according to age (<70 years vs. ≥70 years), ECOG performance-status score (0 or 1 vs. 2), and planned use of combined androgen blockade for more than 30 days (yes vs. no) or agents approved for prevention of skeletal-related events in castration-resistant disease (zoledronic acid or denosumab) (yes vs. no). Patients were also stratified according to the duration of prior adjuvant ADT (<12 months vs. ≥12 months) and the extent of metastases (high volume [defined as the presence of visceral metastases or ≥4 bone lesions with ≥1 beyond the vertebral bodies and pelvis] vs. low volume).
	1.2 Was the allocation sequence concealed until participants were enrolled and assigned to interventions?		

			(Based on direct checks of the IPD) The cumulative number of participants allocated to each intervention group is balanced over time; participants were allocated to a similar degree to each intervention group on each day of the week and there were no weekend randomisations (which is the usual for cancer trials).<br><br>(Based on the manuscript) The sequence was concealed as participants were allocated centrally via a Central Randomisation Desk at the ECOG coordinating centre.
	1.3 Did baseline differences between intervention groups suggest a problem with the randomization process?	N	(Based on direct checks of the IPD) The baseline characteristics age, performance status, disease stage, Gleason score, location of metastases, volume of disease, risk status, number of bone metastases, PSA and BMI were well balanced by intervention group.
	**Risk of bias judgement**	**Low**	
**Bias due to deviations from intended interventions**	2.1.Were participants aware of their assigned intervention during the trial?	Y	As this was a trial of docetaxel chemotherapy plus hormone therapy versus hormone therapy, with different modes of administration and side effects, participants were necessarily aware of the assigned intervention.<br><br>As this was a trial of docetaxel chemotherapy plus hormone therapy versus hormone therapy, with different modes of administration and side effects, carers and those delivering interventions were necessarily aware of the assigned intervention.
	2.2.Were carers and people delivering the interventions aware of participants' assigned intervention during the trial?		
	2.3. If Y/PY/NI to 2.1 or 2.2: Were there deviations from the intended intervention that arose because of the experimental context?	N	We are not aware of any deviations because of the trial context.
	2.4 If Y/PY to 2.3: Were these deviations likely to have affected the outcome?	NA	

	2.5. If Y/PY/NI to 2.4: Were these deviations from intended intervention balanced between groups?	NA	
	2.6 Was an appropriate analysis used to estimate the effect of assignment to intervention?	Y	Using the IPD, we conducted an appropriate intention-to-treat analysis of all randomised participants.
	2.7 If N/PN/NI to 2.6: Was there potential for a substantial impact (on the result) of the failure to analyse participants in the group to which they were randomized?	NA	
	**Risk of bias judgement**	**Low**	
**Bias due to missing outcome data**	3.1 Were data for this outcome available for all, or nearly all, participants randomized?	Y	IPD for progression-free survival were available for all participants randomised.
	3.2 If N/PN/NI to 3.1: Is there evidence that result was not biased by missing outcome data?	NA	
	3.3 If N/PN to 3.2: Could missingness in the outcome depend on its true value?	NA	
	3.4 If Y/PY/NI to 3.3: Is it likely that missingness in the outcome depended on its true value?	NA	
	**Risk of bias judgement**	**Low**	
**Bias in measurement of the outcome**	4.1 Was the method of measuring the outcome inappropriate?	N	Progression-free survival and the events that comprise it (clinical progression, radiological progression and death) are standard and appropriate measures for assessing the effects of treatment on prostate cancer progression. The time to all these events was collected for this trial.
	4.2 Could measurement or ascertainment of the outcome have differed between intervention groups?	PN	This is a composite outcome based on clinical progression, radiological progression or death, whichever occurred first. Measurement of the individual events by intervention group were as follows:

			(Based on the protocol) Clinical progression in this trial is defined as (a) increasing symptomatic bone metastases, (b) clinical deterioration due to cancer per investigator's opinion or (c) progression per RECIST criteria. For the meta-analysis, clinical progression events included events from parts (a) and (b) of the trial clinical progression definition.<br><br>(Based on the protocol) Clinical progression was assessed for all patients at baseline, then every 3 months for up to years; then every 6 months up to 5 years, then yearly thereafter on both arms. Additionally, during docetaxel treatment only, patients on the intervention arm were followed up every 3 weeks.<br><br>Although this outcome is somewhat subjective, awareness of the intervention is unlikely to affect assessment of such clinical symptoms.<br><br>(Based on direct checks of the IPD) Also, as expected, there is good correlation between the incidence of clinical and subsequent radiological progression.<br><br>(Based on the protocol and the manuscript) Radiological progression in this trial was defined as at least a 20% increase in the sum of the longest diameters of target lesions, taking as reference the smallest sum longest diameter recorded since the baseline measurements, or the appearance of one or more new lesion(s); determined according to the Response Evaluation Criteria in Solid Tumors (RECIST), version 1.0) and is unlikely to be influenced by knowledge of the intervention. For the meta-analysis radiological progression events included events from part (c) of the trial clinical progression definition.

			(Based on the protocol) Radiological progression was assessed for all patients at baseline, then at the time of documented castrate-resistant disease (clinical or serologic progression with a testosterone level of less than 50 ng per decilitre; or source documentation of medical castration or surgical castration) or as clinically indicated (confirmed by trialist as either (a) or (b) above).<br><br>(Based on direct checks of the IPD) The duration of follow-up is balanced across intervention groups.
	4.3 Were outcome assessors aware of the intervention received by study participants?	Y	Yes
	4.4 If Y/PY/NI to 4.3: Could assessment of the outcome have been influenced by knowledge of intervention received?	PN	This is a composite outcome based on clinical progression, radiological progression or death, whichever occurred first. It is unlikely that the events that make up the outcome could have been influenced by knowledge of intervention received given the criteria described in 4.2.<br><br>Moreover, (based on direct checks of the IPD), the more objective events dominate this outcome (radiological 80% and deaths 13%) compared to the more subjective events (clinical 7%).<br><br>Assessment of the death could not be influenced by knowledge of the intervention.
	4.5 If Y/PY/NI to 4.4: Is it likely that assessment of the outcome was influenced by knowledge of intervention received?	NA	
	**Risk of bias judgement**	**Low**	
**Bias in selection of**	5.1 Were the data that produced this result analysed in accordance with a pre-specified analysis plan that was finalized before unblinded outcome data were available for analysis?	Y	Our analysis of the trial and the meta-analysis are derived directly from the IPD and not based on reported results. Both follow a statistical analysis plan, specified before the analysis was conducted, unless otherwise stated.

**the reported result**	5.2 ... multiple eligible outcome measurements (e.g. scales, definitions, time points) within the outcome domain?	N	
	5.3 ... multiple eligible analyses of the data?	N	
	**Risk of bias judgement**	**Low**	Our analysis of the trial and the meta-analysis are derived directly from the IPD and not based on reported results. Both follow a statistical analysis plan, specified before the analysis was conducted, unless otherwise stated.
**Overall bias**	**Risk of bias judgement**	**Low**	

Table S1c: Summary of risk of bias assessments for all trials: Failure Free Survival

**Study ID**	**STAMPEDE A vs C (FFS)**		
**Domain**	**Signalling question**	**Response**	**Comments**
**Bias arising from the randomization process**	1.1 Was the allocation sequence random?	Y	(Based on the manuscript) The allocation sequence was generated by minimisation with a random element of 80% stratifying for hospital, age at randomisation, presence of metastases, planned radiotherapy use, nodal involvement, WHO performance status, planned hormone therapy, and regular use of aspirin or another non-steroidal anti-inflammatory drug. NB: Allocation was in a 2:1 ratio to standard of care only (SOC-only), standard of care plus docetaxel (SOC + DOC).<br><br>(Based on direct checks of the IPD) The cumulative number of participants allocated to each intervention group (accounting for 2:1 allocation ratio) was balanced over time; participants were allocated to a similar degree to each intervention group on each day of the week and there were no weekend randomisations (which is the usual for cancer trials).<br><br>(Based on the manuscript) The allocation sequence was concealed as participants were allocated centrally using a computerised algorithm developed and maintained by the MRC CTU.
	1.2 Was the allocation sequence concealed until participants were enrolled and assigned to interventions?		
	1.3 Did baseline differences between intervention groups suggest a problem with the randomization process?	N	(Based on direct checks of the IPD) The baseline characteristics age, performance status, disease stage, Gleason score, location of metastases, volume of disease, risk status, alkaline phosphatase, PSA, and BMI were well balanced by intervention group.
	**Risk of bias judgement**	**Low**	
**Bias due to deviations**	2.1.Were participants aware of their assigned intervention during the trial?	Y	As this was a trial of docetaxel chemotherapy plus hormone therapy versus hormone therapy, with different modes of administration and

**from intended interventions**	2.2.Were carers and people delivering the interventions aware of participants' assigned intervention during the trial?		side effects, participants were necessarily aware of the assigned intervention.<br><br>As this was a trial of docetaxel chemotherapy plus hormone therapy versus hormone therapy, with different modes of administration and side effects, carers and those delivering interventions were necessarily aware of the assigned intervention.
	2.3. If Y/PY/NI to 2.1 or 2.2: Were there deviations from the intended intervention that arose because of the experimental context?	N	We are not aware of any deviations because of the trial context.
	2.4 If Y/PY to 2.3: Were these deviations likely to have affected the outcome?	NA	
	2.5. If Y/PY/NI to 2.4: Were these deviations from intended intervention balanced between groups?	NA	
	2.6 Was an appropriate analysis used to estimate the effect of assignment to intervention?	Y	Using the IPD, we conducted an appropriate intention-to-treat analysis of all randomised participants.
	2.7 If N/PN/NI to 2.6: Was there potential for a substantial impact (on the result) of the failure to analyse participants in the group to which they were randomized?	NA	
	**Risk of bias judgement**	**Low**	
**Bias due to missing outcome data**	3.1 Were data for this outcome available for all, or nearly all, participants randomized?	Y	IPD for failure-free survival were available for all participants randomised.
	3.2 If N/PN/NI to 3.1: Is there evidence that result was not biased by missing outcome data?	NA	
	3.3 If N/PN to 3.2: Could missingness in the outcome depend on its true value?	NA	
	3.4 If Y/PY/NI to 3.3: Is it likely that missingness in the outcome depended on its true value?		

	Risk of bias judgement	Low	
**Bias in measurement of the outcome**	4.1 Was the method of measuring the outcome inappropriate?	N	Failure-free survival and the events that comprise it (biochemical progression, clinical progression, radiological progression and death) are standard and appropriate measures for assessing the effects of treatment on prostate cancer progression. The time to all these events was collected for this trial.
	4.2 Could measurement or ascertainment of the outcome have differed between intervention groups?	PN	It is a composite outcome based on biochemical progression, clinical progression, radiological progression, or death, whichever occurred first. Measurement of the individual events by intervention group were as follows:<br><br>(Based on the protocol) Biological progression in this trial was defined as:<br><br>a. If PSA nadir is more than 50% of the last pre-treatment PSA.<br><br>b. If PSA falls by more than 50% of the last pre-treatment PSA, but remains above 4ng/ml, PSA relapse will be deemed to have occurred when PSA is confirmed as increasing by 50% above the nadir level.<br><br>c. If PSA falls below 4ng/ml, PSA relapse will be defined by either 50% increase from their nadir or the PSA increasing above 4, whichever is the greater. For example, a nadir PSA of 3.6 would require a PSA of 5.4 to define relapse, while PSA nadir of 2.5 will be considered to have relapsed at a PSA of 4.<br><br>(Based on the protocol) Patients on both arms had PSA measured pre-hormone therapy and at weeks 6, 12, 18 and 24 and every 12 weeks, thereafter, up to 2 years post randomisation. Following this, PSA was measured every 6 months until 5 years and annually, thereafter.<br><br>(Based on trial case report forms and information provided by the

			trialists) Clinical progression was not formally defined for this trial. Therefore, as per the meta-analysis data dictionary, clinical progression events comprise any skeletal-related event (SRE), which included bone pain, bone fracture, spinal cord compression documented for this trial.<br><br>(Based on the protocol) Participants were clinically assessed by the investigating physician at each visit: at baseline; weeks 6, 12, 18 and 24; and then every 12 weeks to 2 years; every 6 months to 5 years, then annually on both arms.<br><br>Although this outcome is somewhat subjective, awareness of the intervention is unlikely to affect assessment of such clinical symptoms.<br><br>(Based on direct checks of the IPD) Also, as expected, there is good correlation between the incidence of clinical and subsequent biological and radiological progression.<br><br>(Based on protocol) Radiological progression in this trial was defined as at least a 20% increase in the sum of longest dimension (LD) target lesions taking as reference the smallest sum LD recorded since study entry and/or the appearance of one or more new lesions) and is unlikely to have been influenced by knowledge of the intervention.<br><br>(Based on the protocol and information provided by the trialists) Radiological progression was assessed for all patients at baseline, with a repeat scan advised (but not mandated) at 24 weeks or whenever deemed clinically relevant. Clinically relevant was defined by the trial team as anything that would have triggered the clinician's concern that the patient's cancer was getting worse (e.g., rising PSA, SREs or global general deterioration).

			(Based on direct checks of the IPD) The duration of follow-up is balanced across intervention groups.
	4.3 Were outcome assessors aware of the intervention received by study participants?	Y	Yes
	4.4 If Y/PY/NI to 4.3: Could assessment of the outcome have been influenced by knowledge of intervention received?	PN	This is a composite outcome based on biochemical progression, clinical progression, radiological progression, or death, whichever occurred first. It is unlikely that the events that make up the outcome could have been influenced by knowledge of intervention received given the criteria described in 4.2.<br><br>Moreover, (based on direct checks of the IPD), the more objectively measured events dominate this outcome (biochemical 81%, radiological 9% and deaths 5%) compared to the more subjective events (clinical 5%).<br><br>Additionally, assessment of death could not be influenced by knowledge of the intervention.
	4.5 If Y/PY/NI to 4.4: Is it likely that assessment of the outcome was influenced by knowledge of intervention received?	NA	
	**Risk of bias judgement**	**Low**	
**Bias in selection of the reported result**	5.1 Were the data that produced this result analysed in accordance with a pre-specified analysis plan that was finalized before unblinded outcome data were available for analysis?	Y	Our analysis of the trial and the meta-analysis are derived directly from the IPD and not based on reported results. Both follow a statistical analysis plan, specified before the analysis was conducted, unless otherwise stated.
	5.2 ... multiple eligible outcome measurements (e.g. scales, definitions, time points) within the outcome domain?	N	
	5.3 ... multiple eligible analyses of the data?	N	
	**Risk of bias judgement**	**Low**	

Overall bias	Risk of bias judgement	Low	
**Study ID**	**GETUG-15 (FFS)**		
**Domain**	**Signalling question**	**Response**	**Comments**
**Bias arising from the randomization process**	1.1 Was the allocation sequence random?	Y	(Based on the manuscript) Patients were randomly allocated in a 1:1 ratio to receive ADT plus docetaxel or ADT alone. Dynamic minimisation was used to minimise the imbalance of three criteria: previous systemic treatment with ADT; chemotherapy for local disease or isolated rising PSA; and Glass risk groups<br><br>(Based on direct checks of the IPD) The cumulative number of participants allocated to each intervention group is balanced over time; participants were allocated to a similar degree to each intervention group on each day of the week and there were no weekend randomisations (which is the usual for cancer trials).<br><br>(Based on the manuscript) Treatment allocation was done by a clinical research organisation and was centralised nationally.
	1.2 Was the allocation sequence concealed until participants were enrolled and assigned to interventions?		
	1.3 Did baseline differences between intervention groups suggest a problem with the randomization process?	N	(Based on direct checks of the IPD) The baseline characteristics age, performance status, disease stage, method of diagnosis, Gleason score, location of metastases, volume of disease, risk status, alkaline phosphatase, number of bone metastases, PSA, and BMI were well balanced by intervention group.
	**Risk of bias judgement**	**Low**	
**Bias due to deviations**	2.1.Were participants aware of their assigned intervention during the trial?	Y	As this was a trial of docetaxel chemotherapy plus hormone therapy versus hormone therapy, with different modes of administration and

**from intended interventions**	2.2.Were carers and people delivering the interventions aware of participants' assigned intervention during the trial?	Y	side effects, participants were necessarily aware of the assigned intervention<br><br>As this was a trial of docetaxel chemotherapy plus hormone therapy versus hormone therapy, with different modes of administration and side effects, carers and those delivering interventions were necessarily aware of the assigned intervention.
	2.3. If Y/PY/NI to 2.1 or 2.2: Were there deviations from the intended intervention that arose because of the experimental context?	N	We are not aware of any deviations because of the trial context
	2.4 If Y/PY to 2.3: Were these deviations likely to have affected the outcome?	NA	
	2.5. If Y/PY/NI to 2.4: Were these deviations from intended intervention balanced between groups?	NA	
	2.6 Was an appropriate analysis used to estimate the effect of assignment to intervention?	Y	Using the IPD, we conducted an appropriate intention-to-treat analysis of all randomised participants
	2.7 If N/PN/NI to 2.6: Was there potential for a substantial impact (on the result) of the failure to analyse participants in the group to which they were randomized?	NA	
	**Risk of bias judgement**	**Low**	
**Bias due to missing outcome data**	3.1 Were data for this outcome available for all, or nearly all, participants randomized?	Y	IPD for failure-free survival were available for all participants randomised.
	3.2 If N/PN/NI to 3.1: Is there evidence that result was not biased by missing outcome data?	NA	
	3.3 If N/PN to 3.2: Could missingness in the outcome depend on its true value?	NA	

	3.4 If Y/PY/NI to 3.3: Is it likely that missingness in the outcome depended on its true value?		
	**Risk of bias judgement**	**Low**	
**Bias in measurement of the outcome**	4.1 Was the method of measuring the outcome inappropriate?	N	Failure-free survival and the events that comprise it (biochemical progression, clinical progression, radiological progression, or death) are standard and appropriate measures for assessing the effects of treatment on prostate cancer progression. Although clinical (symptomatic) progression was not collected for this trial; the time to biochemical progression, radiological progression and death were collected.
	4.2 Could measurement or ascertainment of the outcome have differed between intervention groups?	PN	This is a composite outcome based on biochemical progression, radiological progression or death, whichever occurred first. Measurement of the individual events by intervention group were as follows:<br><br>(Based on the protocol) Biochemical progression was defined as:<br><br>a. Patients who have not had a fall in PSA, or a fall of PSA of < 50%, progression<br><br>i. 25% increase in relation to the nadir with a minimum increase in absolute value of 5 ng/ml. Any progression was be confirmed by a second sample.<br><br>b. Patients who have had a response on PSA of > 50% confirmed by a second sample one month later.<br><br>i.an increase of > 50% in relation to the nadir with a minimum increase in absolute value of 5 ng/ml. Any progression was be confirmed by a second sample.

			(Based on the protocol) Patients on both arms had PSA measured at baseline and then every 12 weeks, up to 3.5 years post randomisation. Following this, PSA was be measured every 6 months progression.<br><br>(Based on the protocol) Radiological progression in this trial was defined in the trial as the progression of pre-existing lesions with Response Evaluation Criteria in Solid Tumors (RECIST; version 1.0) or the occurrence of (new) bone lesions, whichever happened first) and is unlikely to have been influenced by knowledge of the intervention.<br><br>(Based on the protocol) Radiological progression was assessed for all patients at baseline and then every 12 weeks for up to 3.5 years; then every 6 months until progression on both arms.<br><br>(Based on direct checks of the IPD) The duration of follow-up is balanced across intervention groups.
	4.3 Were outcome assessors aware of the intervention received by study participants?	Y	Yes
	4.4 If Y/PY/NI to 4.3: Could assessment of the outcome have been influenced by knowledge of intervention received?	PN	This is a composite outcome based on biochemical progression, radiological progression, or death, whichever occurred first. It is unlikely that the events that make up the outcome could have been influenced by knowledge of intervention received given the criteria described in 4.2.<br><br>Moreover, this outcome is solely comprised of the more objective events as clinical (symptomatic) progression was not collected for this trial.<br><br>Additionally, assessment of death could not be influenced by knowledge of the intervention.
	4.5 If Y/PY/NI to 4.4: Is it likely that assessment of the outcome was influenced by knowledge of intervention received?	NA	

	**Risk of bias judgement**	**Low**	
**Bias in selection of the reported result**	5.1 Were the data that produced this result analysed in accordance with a pre-specified analysis plan that was finalized before unblinded outcome data were available for analysis?	Y	Our analysis of the trial and the meta-analysis are derived directly from the IPD and not based on reported results. Both follow a statistical analysis plan, specified before the analysis was conducted, unless otherwise stated
	5.2 ... multiple eligible outcome measurements (e.g. scales, definitions, time points) within the outcome domain?	N	
	5.3 ... multiple eligible analyses of the data?	N	
	**Risk of bias judgement**	**Low**	
**Overall bias**	**Risk of bias judgement**	**Low**	
**Study ID**	**CHAARTED (FFS)**		
**Domain**	**Signalling question**	**Response**	**Comments**
**Bias arising from the randomization process**	1.1 Was the allocation sequence random?	Y	(Based on the protocol) 'The method of permuted blocks will be used for subject randomization. No per-site treatment-allocation balance will be implemented'<br><br>(Based on the manuscript) Patients were stratified according to age (<70 years vs. ≥70 years), ECOG performance-status score (0 or 1 vs. 2), and planned use of combined androgen blockade for more than 30 days (yes vs. no) or agents approved for prevention of skeletal-related events in castration-resistant disease (zoledronic acid or denosumab) (yes vs. no). Patients were also stratified according to the duration of prior adjuvant ADT (<12 months vs. ≥12 months) and the extent of
	1.2 Was the allocation sequence concealed until participants were enrolled and assigned to interventions?		

			metastases (high volume [defined as the presence of visceral metastases or ≥4 bone lesions with ≥1 beyond the vertebral bodies and pelvis] vs. low volume).<br><br>(Based on direct checks of the IPD) The cumulative number of participants allocated to each intervention group is balanced over time; participants were allocated to a similar degree to each intervention group on each day of the week and there were no weekend randomisations (which is the usual for cancer trials).<br><br>(Based on the manuscript) The sequence was concealed as participants were allocated centrally via a Central Randomisation Desk at the ECOG coordinating centre.
	1.3 Did baseline differences between intervention groups suggest a problem with the randomization process?	N	(Based on direct checks of the IPD) The baseline characteristics age, performance status, disease stage, Gleason score, location of metastases, volume of disease, risk status, number of bone metastases, PSA and BMI were well balanced by intervention group.
	**Risk of bias judgement**	**Low**	
**Bias due to deviations from intended interventions**	2.1.Were participants aware of their assigned intervention during the trial?	Y	As this was a trial of docetaxel chemotherapy plus hormone therapy versus hormone therapy, with different modes of administration and side effects, participants were necessarily aware of the assigned intervention.<br><br>As this was a trial of docetaxel chemotherapy plus hormone therapy versus hormone therapy, with different modes of administration and side effects, carers and those delivering interventions were necessarily aware of the assigned intervention.
	2.2.Were carers and people delivering the interventions aware of participants' assigned intervention during the trial?	Y	
	2.3. If Y/PY/NI to 2.1 or 2.2: Were there deviations from the intended intervention that arose because of the experimental context?	N	We are not aware of any deviations because of the trial context.

	2.4 If Y/PY to 2.3: Were these deviations likely to have affected the outcome?	NA	
	2.5. If Y/PY/NI to 2.4: Were these deviations from intended intervention balanced between groups?	NA	
	2.6 Was an appropriate analysis used to estimate the effect of assignment to intervention?	Y	Using the IPD, we conducted an appropriate intention-to-treat analysis of all randomised participants.
	2.7 If N/PN/NI to 2.6: Was there potential for a substantial impact (on the result) of the failure to analyse participants in the group to which they were randomized?	NA	
	**Risk of bias judgement**	**Low**	
**Bias due to missing outcome data**	3.1 Were data for this outcome available for all, or nearly all, participants randomized?	Y	IPD for failure-free survival were available for all participants randomised.
	3.2 If N/PN/NI to 3.1: Is there evidence that result was not biased by missing outcome data?	NA	
	3.3 If N/PN to 3.2: Could missingness in the outcome depend on its true value?	NA	
	3.4 If Y/PY/NI to 3.3: Is it likely that missingness in the outcome depended on its true value?		
	**Risk of bias judgement**	**Low**	
**Bias in measurement of the outcome**	4.1 Was the method of measuring the outcome inappropriate?	N	Failure-free survival and the events that comprise it (biological progression, clinical progression, radiological progression, or death) are standard and appropriate measures for assessing the effects of treatment on prostate cancer progression. The time to all these events was collected for this trial.

	4.2 Could measurement or ascertainment of the outcome have differed between intervention groups?	PN	This is a composite outcome based on biochemical progression, clinical progression, radiological progression, or death, whichever occurred first. Measurement of the individual events by intervention group were as follows:<br><br>(Based on the protocol) Biochemical progression was defined as at least a ≥ 50% increase in PSA level measured from the initiation of therapy taking as reference the lowest PSA level recorded since start of treatment.<br><br>(Based on the protocol) Patients on treatment arm had PSA measured before hormone therapy at baseline, then every 3 weeks while receiving docetaxel, at 6 months and every 3 months thereafter. Patients on control arm had PSA measured before hormone therapy, at time of randomisation and every 3 months until 2 years, then every 6 months up to 5 years, then yearly.<br><br>(Based on the protocol) Clinical progression in this trial is defined as (a) increasing symptomatic bone metastases, (b) clinical deterioration due to cancer per investigator's opinion or (c) progression per RECIST criteria. For the meta-analysis, clinical progression events included events from parts (a) and (b) of the trial clinical progression definition.<br><br>(Based on the protocol) Clinical progression was assessed for all patients at baseline, then every 3 months for up to years; then every 6 months up to 5 years, then yearly thereafter on both arms. Additionally, during docetaxel treatment only, patients on the intervention arm were followed up every 3 weeks.<br><br>Although this outcome is somewhat subjective, awareness of the intervention is unlikely to affect assessment of such clinical symptoms.

			(Based on direct checks of the IPD) Also, as expected, there is good correlation between the incidence of clinical and subsequent biochemical and radiological progression.<br><br>(Based on the protocol and the manuscript) Radiological progression in this trial was defined as at least a 20% increase in the sum of the longest diameters of target lesions, taking as reference the smallest sum longest diameter recorded since the baseline measurements, or the appearance of one or more new lesion(s); determined according to the Response Evaluation Criteria in Solid Tumors (RECIST), version 1.0) and is unlikely to be influenced by knowledge of the intervention. For the meta-analysis radiological progression events included events from part (c) of the trial clinical progression definition.<br><br>(Based on the protocol) Radiological progression was assessed for all patients at baseline, then at the time of documented castrate-resistant disease (clinical or serologic progression with a testosterone level of less than 50 ng per decilitre; or source documentation of medical castration or surgical castration) or as clinically indicated (confirmed by trialist as either (a) or (b) above).<br><br>(Based on direct checks of the IPD) The duration of follow-up is balanced across intervention groups.
	4.3 Were outcome assessors aware of the intervention received by study participants?	Y	Yes
	4.4 If Y/PY/NI to 4.3: Could assessment of the outcome have been influenced by knowledge of intervention received?	PN	This is a composite outcome based on biochemical progression, clinical progression, radiological progression or death, whichever occurred first. It is unlikely that the events that make up the outcome could have been influenced by knowledge of intervention received given the criteria described in 4.2.<br><br>Moreover, (based on direct checks of the IPD), the more objective
	4.5 If Y/PY/NI to 4.4: Is it likely that assessment of the outcome was influenced by knowledge of intervention received?	NA	

			events dominate this outcome (biochemical 65%, radiological 29% and deaths 4%) compared to the more subjective events (clinical 2%).<br><br>Assessment of the death could not be influenced by knowledge of the intervention.
	**Risk of bias judgement**	**Low**	
**Bias in selection of the reported result**	5.1 Were the data that produced this result analysed in accordance with a pre-specified analysis plan that was finalized before unblinded outcome data were available for analysis?	Y	Our analysis of the trial and the meta-analysis are derived directly from the IPD and not based on reported results. Both follow a statistical analysis plan, specified before the analysis was conducted, unless otherwise stated.
	5.2 ... multiple eligible outcome measurements (e.g. scales, definitions, time points) within the outcome domain?	N	
	5.3 ... multiple eligible analyses of the data?	N	
	**Risk of bias judgement**	**Low**	
**Overall bias**	**Risk of bias judgement**	**Low**	

Supplementary Tables

Table S2: Sensitivity analyses: results for OS PFS and FFS

	Overall Survival				Progression Free survival				Failure Free survival			
	HR	95% CI	P-value	I^2	HR	95% CI	P-value	I^2	HR	95% CI	P-value	I^2
**Fixed effect, unadjusted**	0.80	0.72-0.89	0.000087	0%	0.71	0.64-0.79	6.0×10^{-11}	0%	0.67	0.61-0.73	5.0×10^{-17}	0%
**Random effects, adjusted**	0.79	0.70-0.89	0.00015	13%	0.70	0.63-0.78	4.7×10^{-10}	15%	0.64	0.58-0.72	2.4×10^{-15}	20%
**Fixed effects, complete-case adjusted**	0.76	0.67-0.85	0.0000058	22%	0.69	0.62-0.77	1.6×10^{-11}	0%	0.64	0.58-0.71	7.9×10^{-18}	0%

Table S3: Relative and absolute effects of docetaxel on sensitivity outcomes

**Outcome**	**Relative effect (Fixed effect, adjusted)**		**Absolute effect at 5 years**	
	**HR (95% CI)**	**I^2, heterogeneity p value**	**% Benefit (95% CI)**	**Change from baseline**
Radiological progression-free survival	0.71 (0.64, 0.79), $p=1.2x10^{-10}$	I^2=0%, p=0.52	9% (6-13%)	26% to 35%
Prostate cancer specific survival	0.78 (0.69, 0.88), p=0.000062	I^2=0%, p=0.44	10% (6-14%)	46% to 55%.
Time to PSA failure	0.60 (0.53, 0.67), $p=2.0x10^{-20}$	I^2=0%, p=0.72	10% (6-13%)	16% to 26%.
Time to castrate-resistant disease	0.61 (0.56, 0.68), $p=3.9x10^{-22}$	I^2=0%, p=0.48	10% (7-14%)	15% to 26%.

Table S4: Effect of docetaxel on PFS by other participant characteristics

	Events / patients	Interaction HR	95% CI	Het. p-value	I^2
**Age**	1624/2261	1.01	0.89-1.15	0.84	0%
**BMI**	1542/2148	0.99	0.79-1.25	0.95	0%
**WHO performance status**	1609/2242	0.91	0.71-1.16	0.93	0%
**Gleason score**	1457/2039	0.90	0.70-1.15	0.41	11%
**Risk Group**	1147/1597	0.82	0.63-1.06	0.12	65%
**Nodal Involvement**	1168/1617	1.00	0.77-1.28	0.97	70%

Table S5. Mutually-adjusted two-way interactions between volume, timing of metastases and clinical T-stage treatment

Covariate	Subgroup comparison	Likelihood-ratio test for interaction between subgroup and treatment effects*	Interaction effect (ratio of HRs) (95% CI)*
Volume of disease	High vs Low	χ^2 = 3.20 on 1 d.f., p=0.073	0.80 (0.63-1.02)
Timing of metastatic disease	Synchronous vs Metachronous	χ^2 = 0.56 on 1 d.f., p=0.45	0.89 (0.64-1.22)
cT Stage	cT3 vs 1-2	χ^2 = 2.14 on 1 d.f., p=0.14	0.80 (0.60-1.08)
	cT4 vs 1-2	χ^2 = 11.9 on 1 d.f., p=0.00055	0.54 (0.38-0.76)
	Joint test	χ^2 = 12.2 on 2 d.f., p=0.0022	

* mutually adjusted for other Ratios of HRs in this Table, and for core adjustment covariates

Table S6. Exploratory analysis showing the effect of docetaxel on PFS

Subgroup	Pooled baseline survival	Treatment effect (95% CI)
Low volume / Metachronous	0.480 (0.373, 0.619)	-0.007 (-0.170, 0.157)
Low volume / Synchronous	0.414 (0.359, 0.478)	0.056 (-0.034, 0.146)
High volume / Metachronous	0.128 (0.068, 0.241)	0.078 (-0.063, 0.218)
Low vol. cT1-3	0.400 (0.351, 0.456)	0.063 (-0.019, 0.145)
Low vol. cT4	0.239 (0.155, 0.368)	0.129 (-0.057, 0.315)
High vol. cT1-3	0.123 (0.099, 0.151)	0.075 (0.028, 0.122)
High vol. cT4	0.077 (0.043, 0.137)	0.245 (0.142, 0.348)

Table S7. Comparison of baseline characteristics of patients by availability of cT stage data

		T stage available (N=1789)		T stage unavailable (N=472)	
**Age years**	**Median (IQR)**	1789/1789	65 (59-70)	472 /472	63 (58-69)
***Alkaline phosphatase IU/l**	**Median (IQR)**	1044/1789	110 (73-246)	178/472	237 (133-562)
**PSA ng/ml**	**Median (IQR)**	1787/1789	47 (10-183)	469/472	41 (6-210)
**ECOG Performance status (n, %)**	0	1167 (65%)		173 (37%)	
	1-2	428 (24%)		108 (23%)	
	Missing	194 (11%)		191 (41%)	
**Risk of disease (n, %)**	Low	586 (33%)		139 (29%)	
	High	699 (39%)		173 (37%)	
	Missing	504 (28%)		160 (34%)	
**Gleason Sum Score (n, %)**	<8	463 (26%)		144 (31%)	
	>=8	1,193 (67%)		239 (51%)	
	Missing	133 (7%)		89 (19%)	
**Nodal involvement (n, %)**	N0	603 (34%)		53 (11%)	
	N+	895 (50%)		66 (14%)	
	Missing	291 (16%)		353 (75%)	
**Timing of diagnosis (n, %)**	Synchronous	1,526 (85%)		357 (76%)	
	Metachronous	260 (15%)		112 (24%)	
	Missing	3 (<1%)		3 (1%)	
**Bone metastases (n, %)**	No	189 (11%)		50 (11%)	
	Yes	1,379 (77%)		363 (77%)	
	Missing	221 (12%)		59 (13%)	
**Visceral metastases (n, %)**	No	1,545 (86%)		393 (83%)	
	Yes	244 (14%)		78 (17%)	
	Missing	0 (0%)		1 (<1%)	
**†Volume of disease (n, %)**	Low	654 (37%)		160 (34%)	
	High	901 (50%)		277 (59%)	
	Missing	234 (13%)		35 (7%)	

Data are median (IQR) or n (%). Percentages might not sum to 100 as a result of rounding. Data supplied for inclusion in the meta-analysis are in keeping with the most recent version of reported trial analysis, as cited.

*In GETUG-15, specific values were only recorded for patients who were noted as having values outside the normal range at randomisation; 219 participants whose baseline reading was recorded as within the normal range had no specific value noted and are included here as missing.

†277 patients in CHAARTED were recorded as low volume without specifically characterising presence or absence of bone metastases.

§Visceral metastases located in lung, liver, or adrenal gland.

Supplementary Figures

Figure S1: Prisma flow diagram

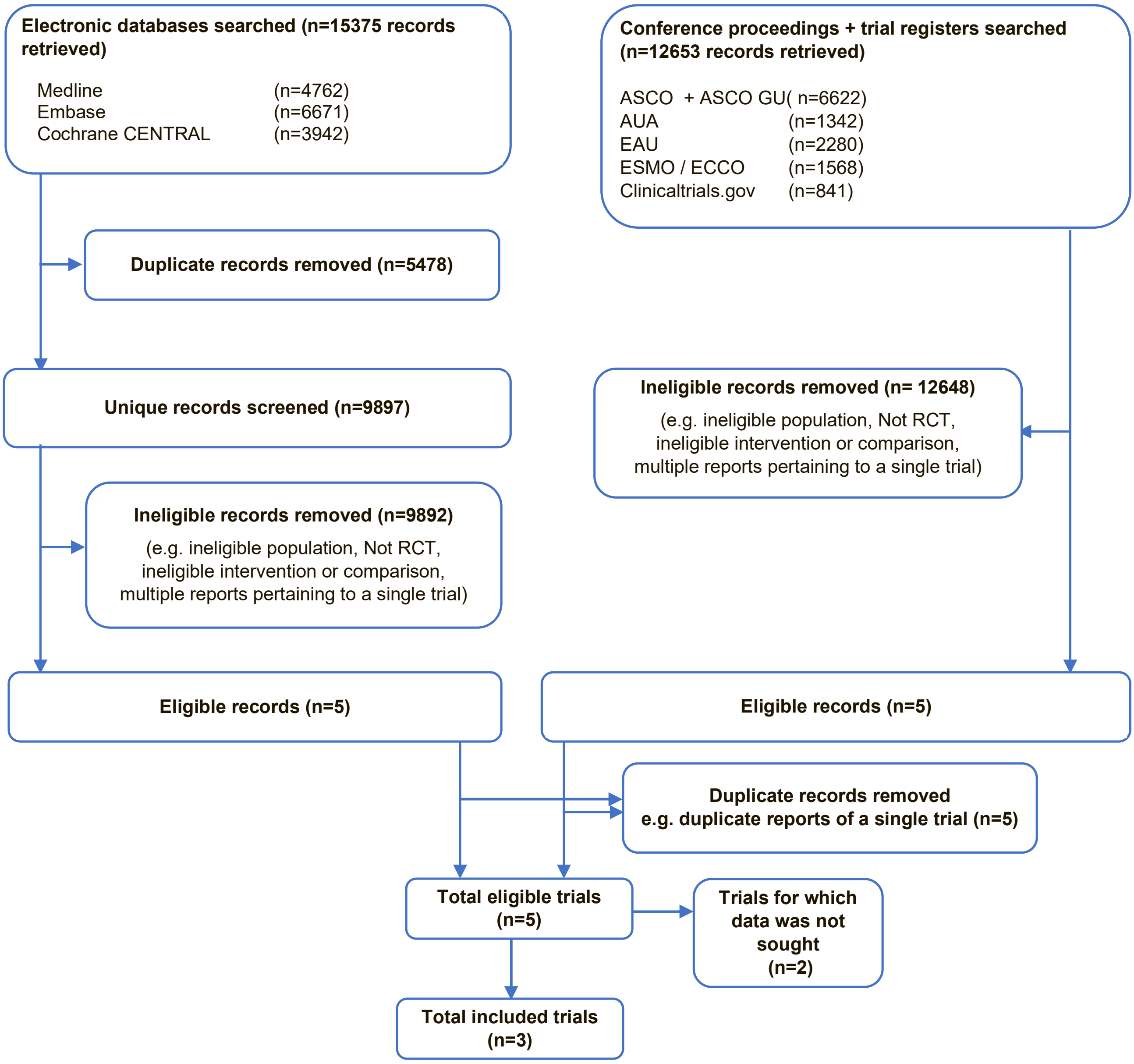

Figure S2: Risk of Bias summary of assessments (traffic light)

a) Overall survival

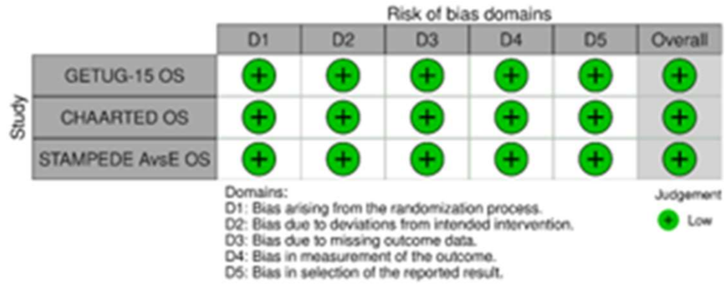

b) Progression-free survival

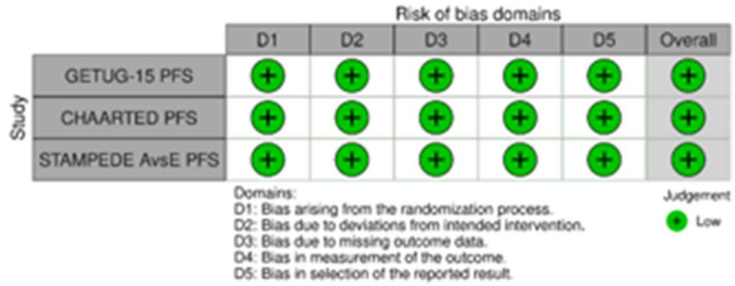

c) Failure-free survival

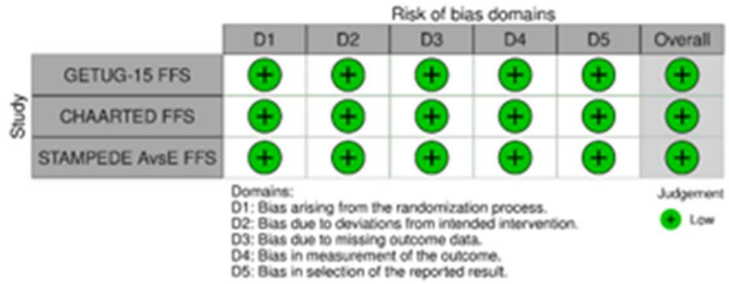

Figure S3: Assessment of proportional hazards for (a) OS, (b) PFS and (c) FFS

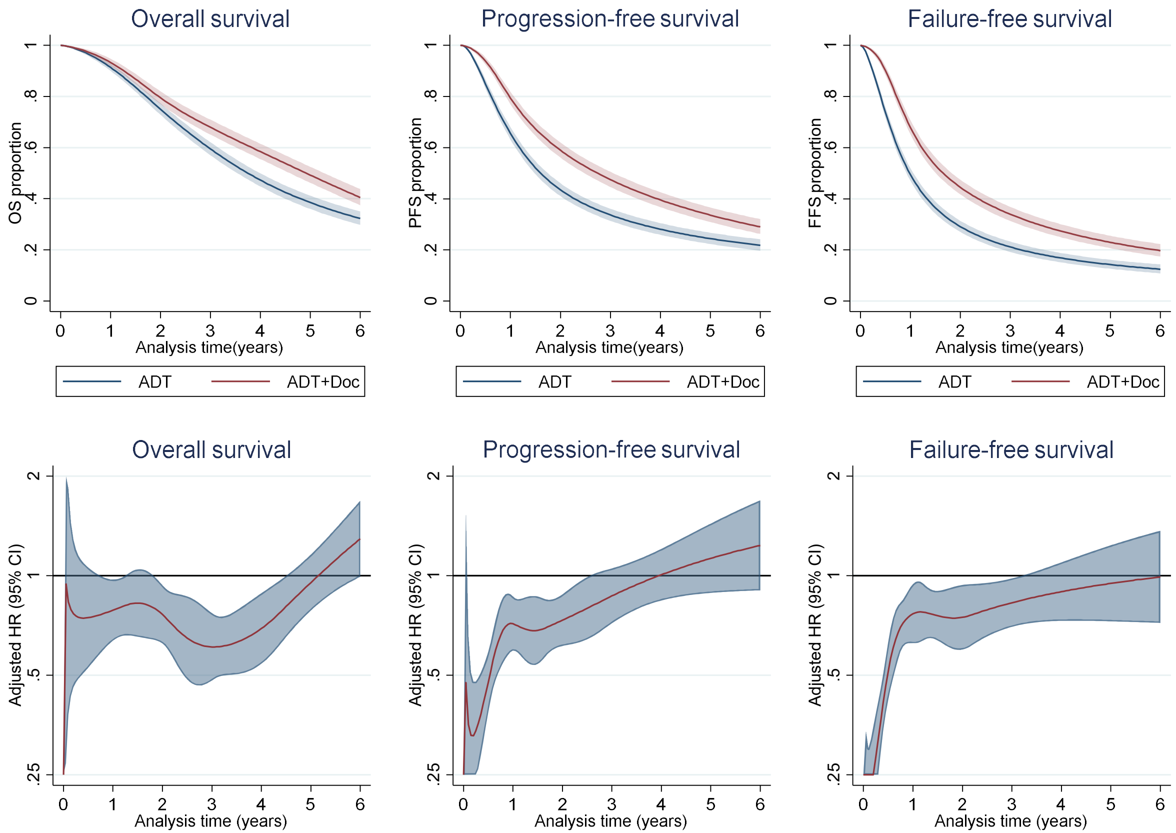

Adjusted for Trial, and for core covariate set (so covariates are harmonized across trials) with missing data imputed. Both plots for each outcome are derived from a single flexible parametric model, with time-varying effects placed on Treatment and Trial. Kaplan-Meier curves are marginalized over observed covariate values (using "standsurv" in Stata) HR plots use predicted values from the flexible parametric model. These HRs should only be used to assess the non-proportionality relationship; they should ***not*** be interpreted as treatment effects.

Figure S4: Overall effects of Docetaxel for OS, PFS FFS

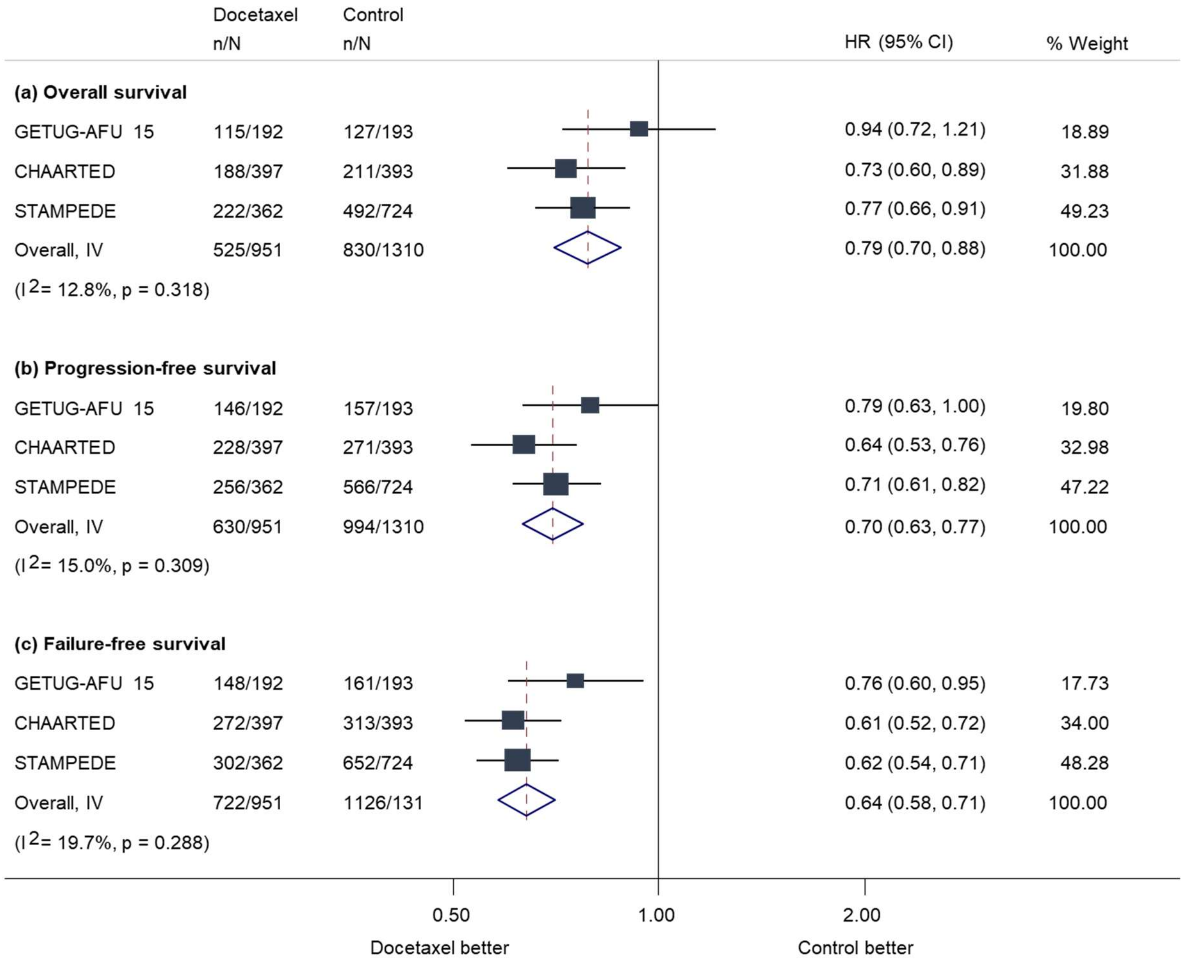

Boxes represent hazard ratios (HRs) derived from Cox regression models fitted to each trial in turn, adjusted for the core covariate set and with missing covariate values imputed. The size of each square is directly proportional to the amount of information contributed by a trial, and the horizontal lines show the 95% confidence interval (CI). The diamonds represent the two-stage, fixed-effect, inverse-variance meta-analysis, with the centre denoting the HR and the extremities the 95% CI.

Figure S5: Absolute effects -Forest plots for OS, PFS FFS

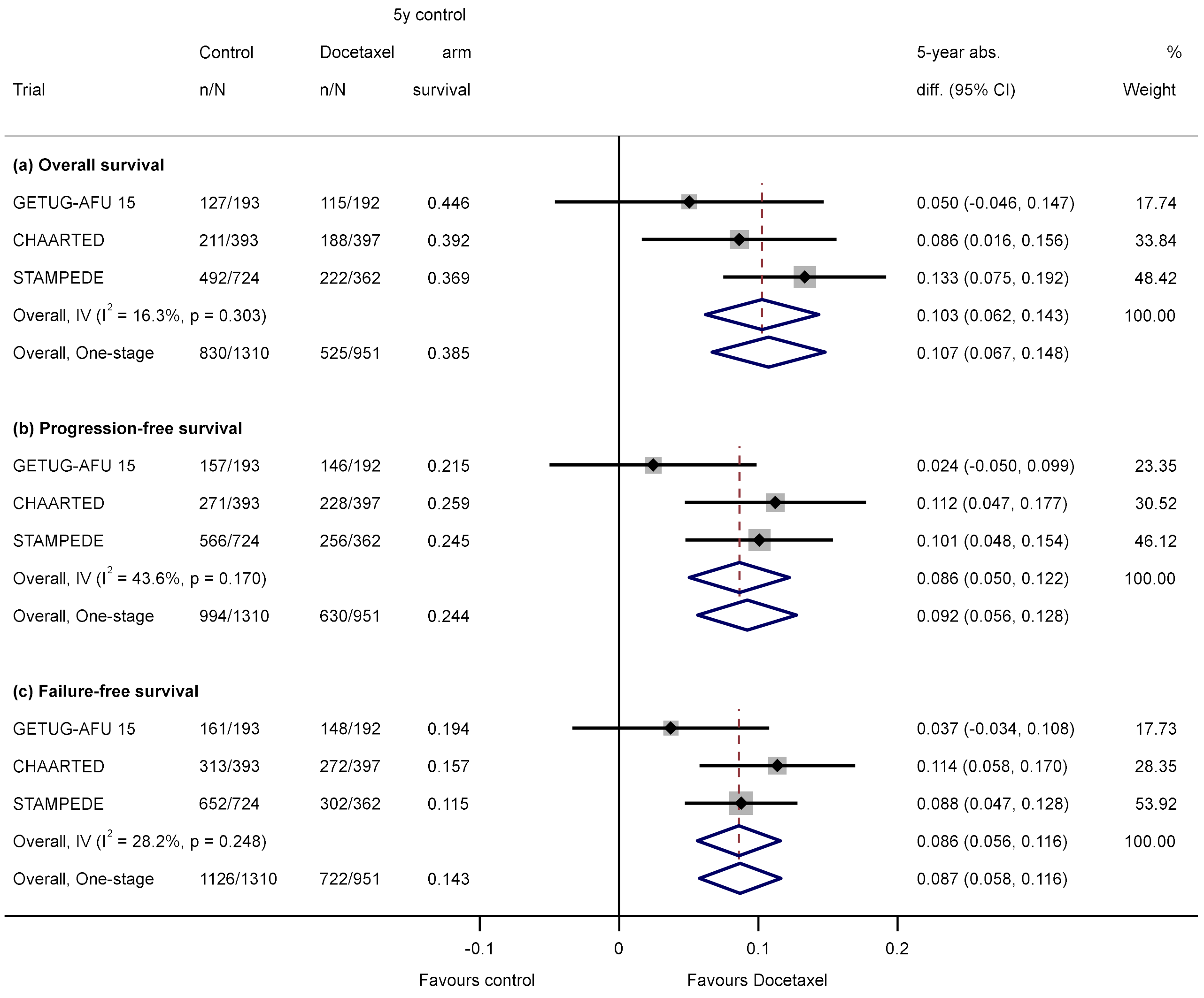

Absolute effects of ADT plus docetaxel versus ADT at 5 years alone overall and by trial for: (A) Overall survival; (B) Progression free survival and (C) Failure free survival.

Figure S6: Flow diagram of progression free survival events

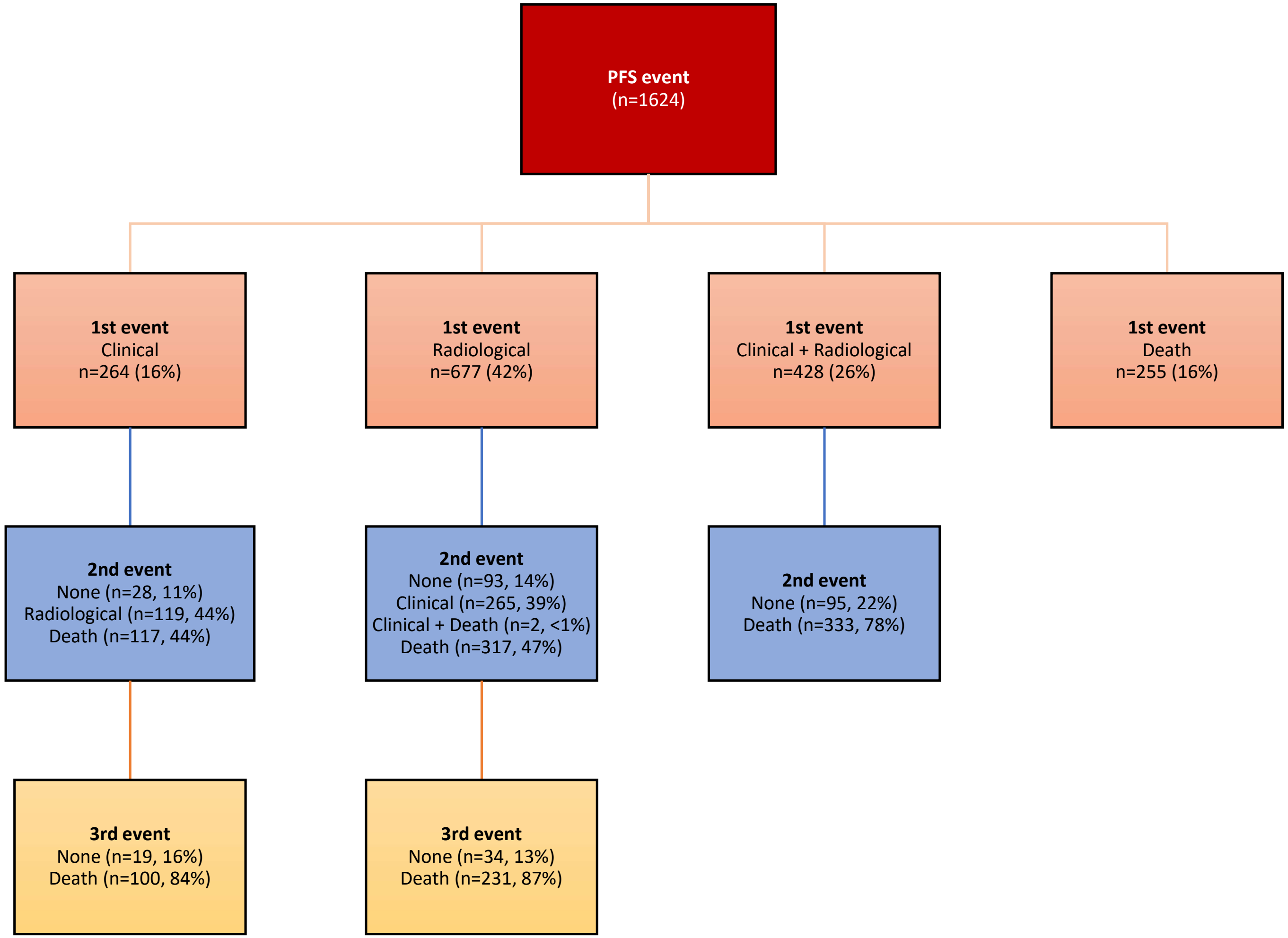

Figure S7: Flow diagram of failure free survival events

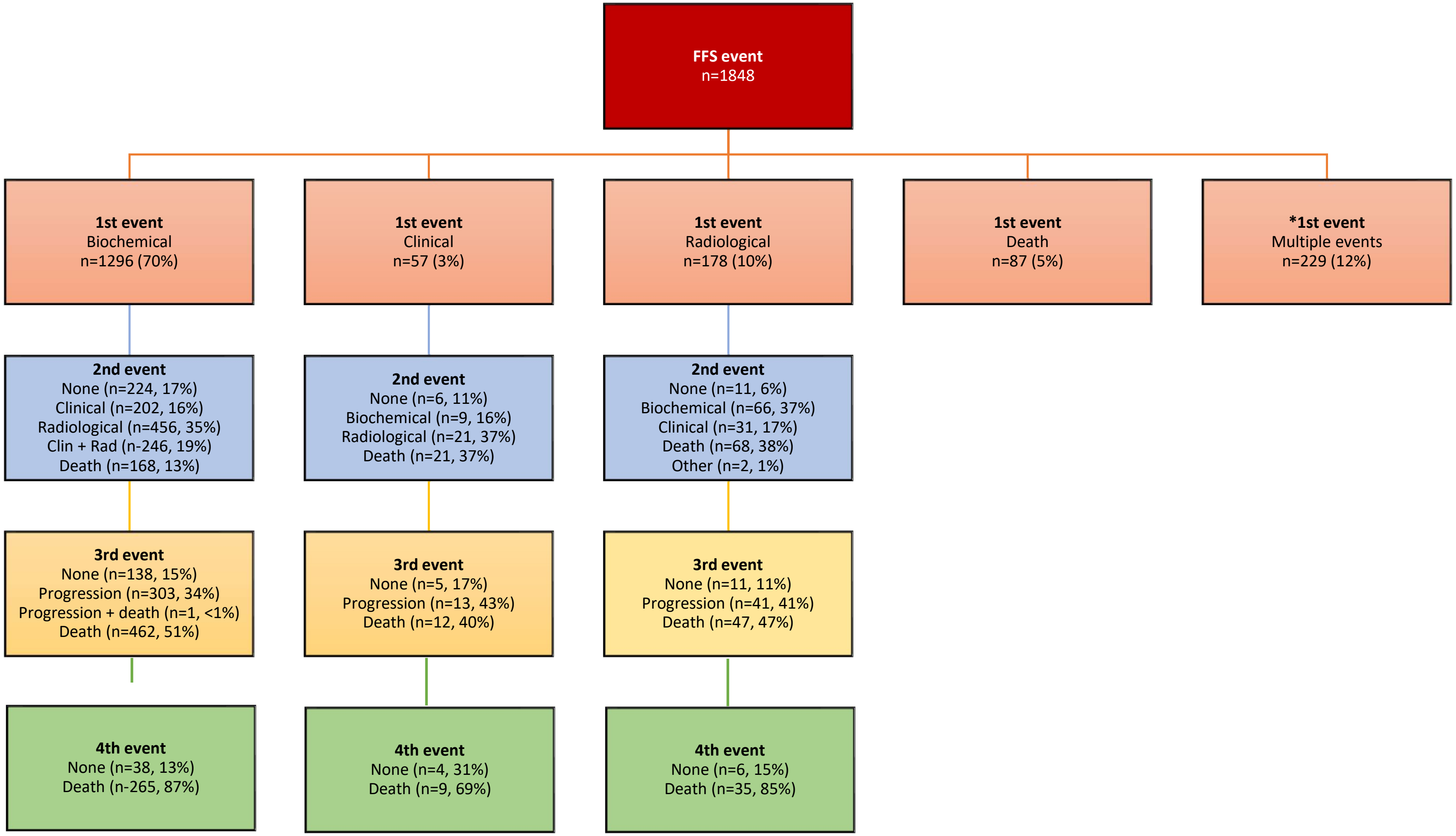

*Of which, 52 events included biochemical progressions in combination with either clinical, radiological or both

Figure S8: Effect of docetaxel on PFS and OS for patients with high volume disease, by timing of metastatic disease diagnosis

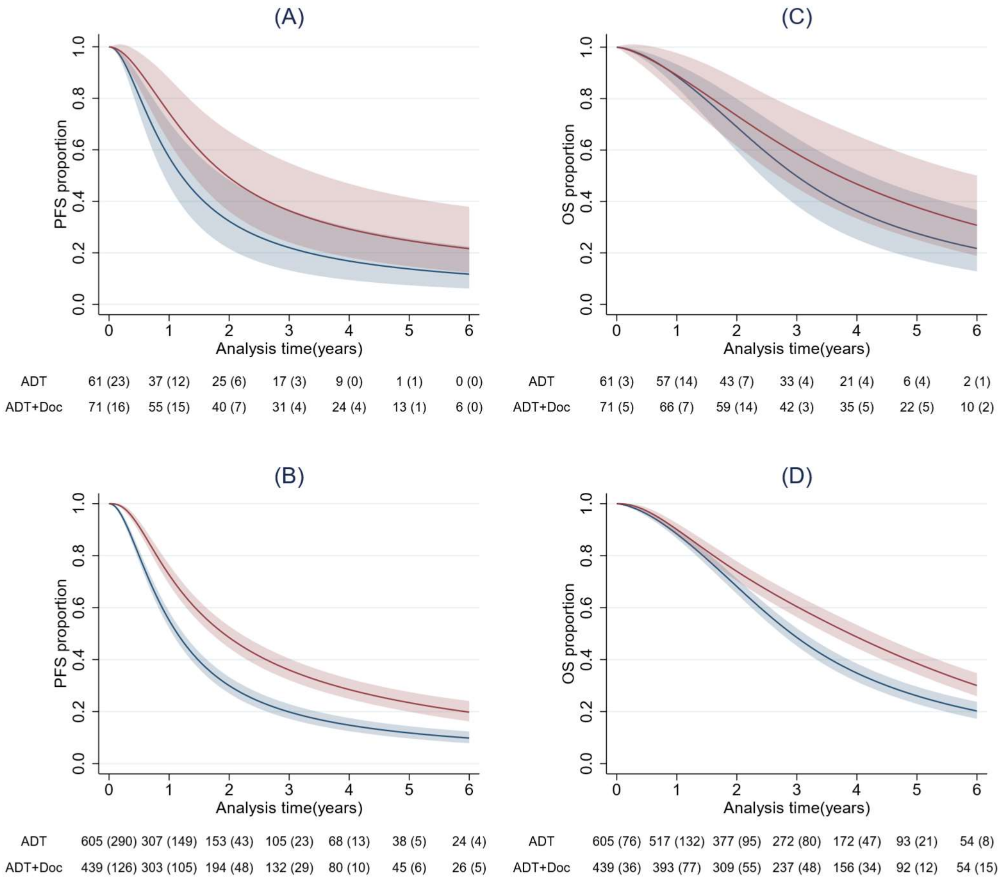

Predicted survival curves for patients with high volume metastatic disease for the subgroups of synchronous and metachronous diagnosis, based on a one-stage flexible parametric meta-analysis model fitted to the entire participant sample with interaction terms between docetaxel effect and each of the four volume-by-timing subgroups, accounting appropriately for aggregation bias, adjusted for the core covariate set and with missing covariate values imputed, and using regression standardization to estimate marginal survival curves for (A) high volume, metachronous disease (PFS); (B) high volume, synchronous disease (PFS); (C) high volume, metachronous disease (OS) and (D) high volume, synchronous disease (OS). Shaded areas denote the 95% confidence intervals for each treatment arm

Figure S9: Effect of docetaxel on PFS and OS for patients with low volume disease, by clinical T stage

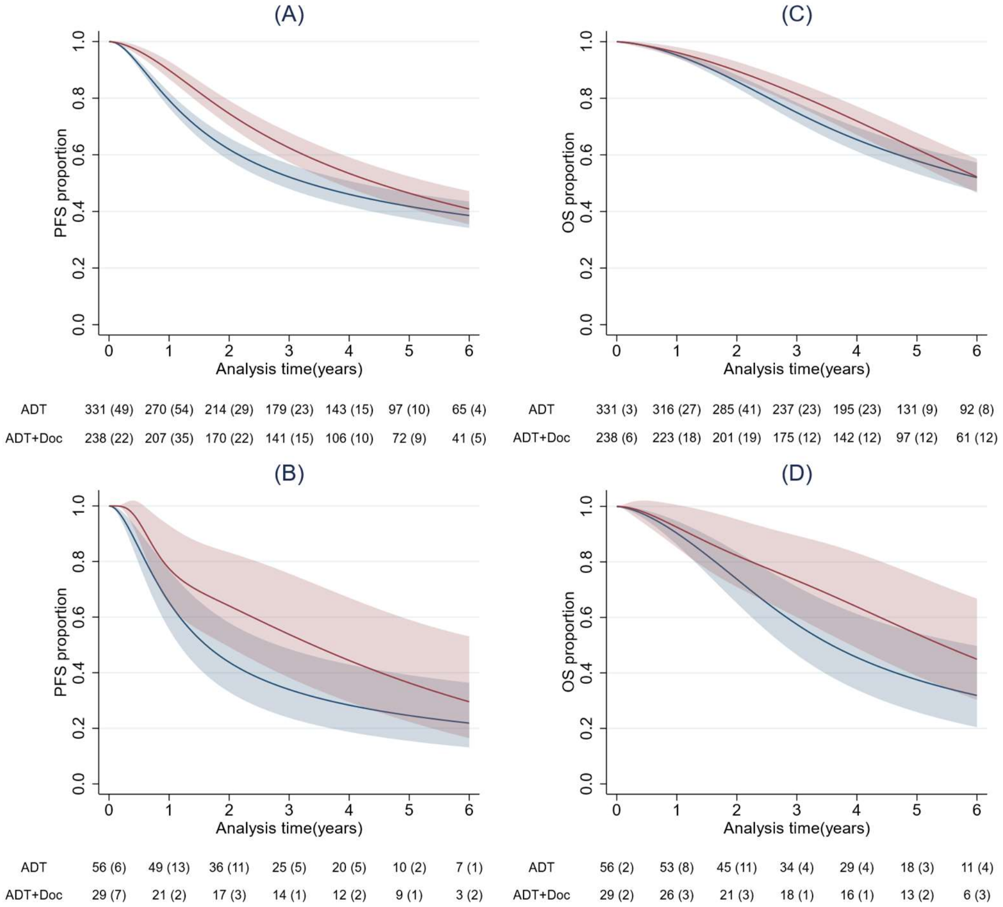

Predicted survival curves for patients with low volume metastatic disease, for subgroups based on clinical T-stage (cT stage), based on a one-stage flexible parametric meta-analysis model fitted to the entire participant sample with interaction terms between docetaxel effect and each of the four volume-by-stage subgroups, accounting appropriately for aggregation bias, adjusted for the core covariate set and with missing covariate values imputed, and using regression standardization to estimate marginal survival curves for (A) low volume, cT stage 1-3 (PFS), (B) low volume, cT stage 4 (PFS), (C) low volume, cT stage 1-3 (OS) and (D) low volume, cT stage 4 (OS). Shaded areas denote the 95% confidence intervals for each treatment arm.
